# High-rate and selective conversion of CO_2_ from aqueous solutions to hydrocarbons

**DOI:** 10.1038/s41467-023-38963-y

**Published:** 2023-06-01

**Authors:** Cornelius A. Obasanjo, Guorui Gao, Jackson Crane, Viktoria Golovanova, F. Pelayo García de Arquer, Cao-Thang Dinh

**Affiliations:** 1 Department of Chemical Engineering, Queen’s University, Kingston, ON K7L 3N6 Canada; 2 ICFO–Institut de Ciències Fotòniques, The Barcelona Institute of Science and Technology, Barcelona, 08860 Spain

**Keywords:** Energy storage, Electrocatalysis, Electrochemistry

## Abstract

Electrochemical carbon dioxide (CO_2_) conversion to hydrocarbon fuels, such as methane (CH_4_), offers a promising solution for the long-term and large-scale storage of renewable electricity. To enable this technology, CO_2_-to-CH_4_ conversion must achieve high selectivity and energy efficiency at high currents. Here, we report an electrochemical conversion system that features proton-bicarbonate-CO_2_ mass transport management coupled with an in-situ copper (Cu) activation strategy to achieve high CH_4_ selectivity at high currents. We find that open matrix Cu electrodes sustain sufficient local CO_2_ concentration by combining both dissolved CO_2_ and in-situ generated CO_2_ from the bicarbonate. In-situ Cu activation through alternating current operation renders and maintains the catalyst highly selective towards CH_4_. The combination of these strategies leads to CH_4_ Faradaic efficiencies of over 70% in a wide current density range (100 – 750 mA cm^-2^) that is stable for at least 12 h at a current density of 500 mA cm^-2^. The system also delivers a CH_4_ concentration of 23.5% in the gas product stream.

## Abstract (web summary)

Electrochemical CO2 conversion to methane offers a promising solution for the large-scale storage of renewable electricity, yet the catalytic selectivity at high current density still needs to be refined. Here the authors report to use both dissolved CO2 and in-situ generated CO2 from bicarbonate to sustain high local CO2 concentration around Cu electrode and thus achieve selective CO2 conversion to methane.

## Introduction

Electrochemical carbon dioxide (CO_2_) reduction (ECR) to chemical fuels offers a large-scale solution to the long-term storage of renewable electricity^1^. Among many possible ECR products, methane (CH_4_) is an appealing target for energy storage applications because of its high energy density (55.5 MJ kg^−1^), widespread use, and large market size (responsible for ~23% of the global energy use)^2^. CH_4_ produced from CO_2_ and renewable electricity could be readily integrated into the existing natural gas infrastructure, providing a direct path to its decarbonization^3^.

Practical ECR systems must operate at high current densities (often >300 mA cm^−2^) with high selectivity and energy efficiency^1,4^. To reduce separation costs, high product concentrations and avoiding CO_2_ loss to carbonate crossover are desirable^5^. Gas-phase ECR systems, including both flow-cell and membrane electrode assembly (MEA) configurations, have been used as platforms to achieve selective CO_2_ conversion at high current densities^6–10^. Using gas-phase systems, CO_2_-to-CH_4_ conversion with high Faradaic efficiencies (FEs) (60–70%) at relatively high current densities (200–300 mA cm^−2^) have been demonstrated^11–22^. However, gas-phase systems often use alkaline electrolytes or anion exchange membranes to achieve these high selectivities. In these configurations, carbonate formation (with alkaline electrolytes) or crossover (with anion exchange membranes) is significant, requiring additional energy to recycle the electrolyte or CO_2_^5,23,24^.

Carbonate and bicarbonate-fed systems have recently been developed to integrate CO_2_ capture and conversion steps within a single system^25–28^. In this architecture, CO_2_ is generated in situ inside the reactor when protons generated from a bipolar membrane (BPM) react with (bi)carbonate. This system offers effective carbon utilization that bypasses the energy-intensive step of extracting CO_2_ from a CO_2_ captured solution^25,29^. It also allows the production of gas products with high concentrations, as the gaseous products do not mix with the CO_2_ feedstock^29^. Using (bi)carbonate fed systems, high carbon monoxide (CO) and formate selectivity (FEs over 70%) at relatively high partial current densities (70–150 mA cm^−2^) have been demonstrated^25,26,28,30^.

Selectivity for CH_4_ production from recent bicarbonate-fed systems is relatively low compared to gas-phase systems, however. The current state-of-the-art for CH_4_ production from bicarbonate exhibited a FE of 30% and a partial current density of 120 mA cm^−2^ ref. ^27^. We hypothesized that this limited performance stems from a yet uncontrolled reaction environment, including both local CO_2_ concentration and local pH, and the lack of selective catalysts, possibly because Cu reconstruction at high current density typically favors the hydrogen evolution reaction^31–34^.

In this work, we unveil the critical role of electrode pore size on local CO_2_ availability in bicarbonate-fed systems using BPMs. We found that large Cu pores enhance the transport of dissolved CO_2_ and favor bicarbonate conversion into CO_2_ leading to high local CO_2_ concentration. We further develop an in-situ catalyst activation strategy that generates and maintains highly specific Cu surfaces for selective CH_4_ production from CO_2_ dissolved in aqueous solutions. We implement this catalyst in large pore electrodes to report selective and concentrated CH_4_ generation, at high current densities. Our aqueous-fed system achieves a CO_2_-to-CH_4_ conversion with over 70% FE at a wide current density range of 100–750 mA cm^−2^, with a record-high CH_4_ partial current density of over 500 mA cm^−2^. The system is also stable, maintaining its high CH_4_ FE for at least 12 h at a current density of 250–500 mA cm^−2^. Our aqueous-fed system also outperforms previous systems in terms of full-cell energy efficiency and delivers a record CH_4_ concentration of up to 23.5% in the gas outlet stream.

## Results

### Modeling the reaction environment and CO_2_ availability

**Fig. 1:**
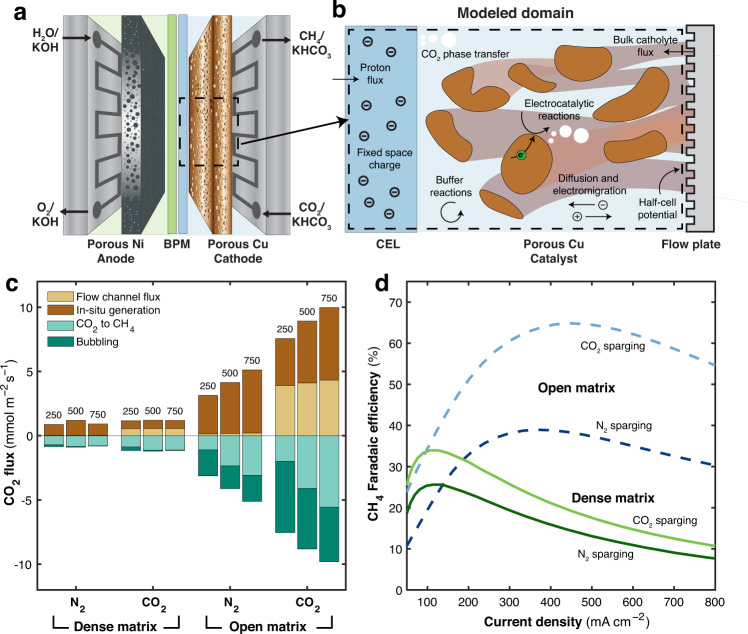
Modeling reaction environment and CO_2_ availability. **a** Schematic illustration of the aqueous solution-fed ECR system using porous Cu cathode, Ni foam anode and bipolar membrane (BPM). **b** Schematic of modeled domain with key physics annotated. **c** CO_2_ flux components at three current densities, 250, 500, and 750 mA cm^−2^ for dense matrix and open matrix catalysts with N_2_ sparging or CO_2_ sparging. 0.3 M KHCO_3_ was used as an electrolyte for all modeling. **d** Modeled CH_4_ FE as a function of current density for a dense matrix catalyst (solid lines) and open matrix catalyst (dashed lines) with CO_2_ sparging and N_2_ sparging.

Electrochemical CO_2_ reduction relies on high CO_2_ availability within the catalyst domain. To study the role of CO_2_ availability in a bicarbonate-fed ECR system, we used one-dimensional multiphysics modeling. The cation exchange layer and porous copper catalyst sub-systems are considered in the model (Fig. 1a, b). Key physics are considered including species and charge transport, electrocatalytic reactions, CO_2_ phase transfer, and buffer equilibrium reactions, with constants derived from past work^35–39^. A proton flux from water dissociation within the BPM, proportional to the total integrated current density, is imposed on the anion exchange layer/cation exchange layer interface. A mass flux boundary condition is imposed on the catalyst layer/flow channel interface from the bulk electrolyte. In this system, CO_2_ comes from two sources: dissolved CO_2_ in the KHCO_3_ electrolyte and CO_2_ generated during the reaction. Under reverse bias, protons (H^+^) generated from the surface of the BPM react with bicarbonate ions (HCO_3_^−^) in the electrolyte, forming CO_2_ in the solution.

1 $${{{{{{{\rm{HCO}}}}}}}_{3}}^{-}+{{{{{{\rm{H}}}}}}}^{+}\to {{{{{{\rm{CO}}}}}}}_{2}+{{{{{{\rm{H}}}}}}}_{2}{{{{{\rm{O}}}}}}$$

The CO_2_ formed in situ diffuses to the surface of the catalyst, and when formed in excess of the solubility limit, forms bubbles on the surface of the membrane (Fig. 1b). CH_4_ is generated in an 8-electron reaction, consuming CO_2_ and producing OH^−^.

2 $${{{{{{\rm{CO}}}}}}}_{2}+{{{{{{\rm{4H}}}}}}}_{2}{{{{{\rm{O}}}}}}+8{{{{{{\rm{e}}}}}}}^{-}\to {{{{{{\rm{CH}}}}}}}_{4}+{{{{{{\rm{8OH}}}}}}}^{-}$$

State-of-the-art bicarbonate electrolyzers rely on catalysts within a dense porous matrix with the CO_2_ source in-situ generation from bicarbonate^26–28^.

Due to the critical importance of CO_2_ availability on electrolyzer performance^40^, we examined CO_2_ availability in the catalyst domain for a dense porous matrix catalyst. We found that most CO_2_ in-situ generation via Eq. 1 occurs near the catalyst-membrane interface (Fig. S1), while dissolved CO_2_ from the bulk electrolyte enters via the flow channel. To generate substantial quantities of C_1+_ products via ECR, CO_2_ must readily diffuse from the boundaries into the catalyst domain. The model is used to perform a detailed CO_2_ accounting at different current densities to understand CO_2_ dynamics within the system. CO_2_ fluxes are sub-divided into four components: (1) flux from the flow channel, (2) in-situ generation (although CO_2_ can also be consumed, on net, via fixing into (bi)carbonate), (3) conversion of CO_2_ to CH_4_, and (4) aqueous to vapor phase transfer advected to the flow channel via bubbling. In the dense matrix catalysts with N_2_ sparging at high current density, in-situ CO_2_ generation is severely restricted, while flow-channel flux is nearly non-existent (Fig. 1c), leading to low CH_4_ selectivity (Fig. 1d).

Given the dominant role of CO_2_ diffusivity on ECR rates, we hypothesized that an open matrix design, which leads to higher diffusion and boundary mass transfer, would improve performance. Modeling suggests that when switching from a dense matrix to an open matrix, both with N_2_ sparging, performance at high current densities improves substantially (Fig. 1d). For example, at 500 mA cm^−2^ total current density, CH_4_ FE increases from 13% to 37% when switching from the dense to the open matrix catalyst. Mechanistically, modeling suggests that high diffusivity from the open matrix facilitates much higher in-situ CO_2_ generation (Fig. 1c). The CO_2_ transport from the flow channel in the open matrix N_2_ sparging case remains, however, low. This leads to adequate CO_2_ availability on the membrane side of the catalyst domain, but poor CO_2_ availability on the flow-channel side (Fig. S2). We further hypothesized that it would be possible to improve the utilization of the entire catalyst domain by increasing CO_2_ concentration from the flow channel by sparging the electrolyte with CO_2_ instead of N_2_. CO_2_ flux tracking shows that when sparging with CO_2_, the CO_2_ influx from the flow channel improves substantially, leading to CH_4_ FEs of up to 65% at 500 mA cm^-2^. Meanwhile, CO_2_ sparging makes only a minor difference in the dense matrix cases (Fig. 1d) presumably because the low diffusivity and mass transport prevent CO_2_ from adequately penetrating the domain. Similar trends are observed for other catholyte concentrations from 0.1 to 1 M KHCO_3_ (Fig. S3).

These results suggest a path forward and design principles to realize bicarbonate-fed electrolyzers with increased performance: a highly porous matrix enables efficient utilization of both dissolved CO_2_ from electrolyte and in-situ formed CO_2_ from bicarbonate.

### In-situ generation of selective catalysts

To implement the open matrix strategy, we selected a cathode consisting of a copper mesh with large pores (average pore diameter of 150 µm and average Cu wire of 100 µm) and a pore density of 100 pores per inch (Fig. S4). Electrochemical CO_2_ reduction was performed using an aqueous-fed system coupled with a BPM operated in reverse bias mode (Fig. S5). A 0.3 M KHCO_3_ solution saturated with CO_2_ was used as both the electrolyte and CO_2_ source and a 1 M KOH solution was used as the anolyte (Fig. 1a).

**Fig. 2:**
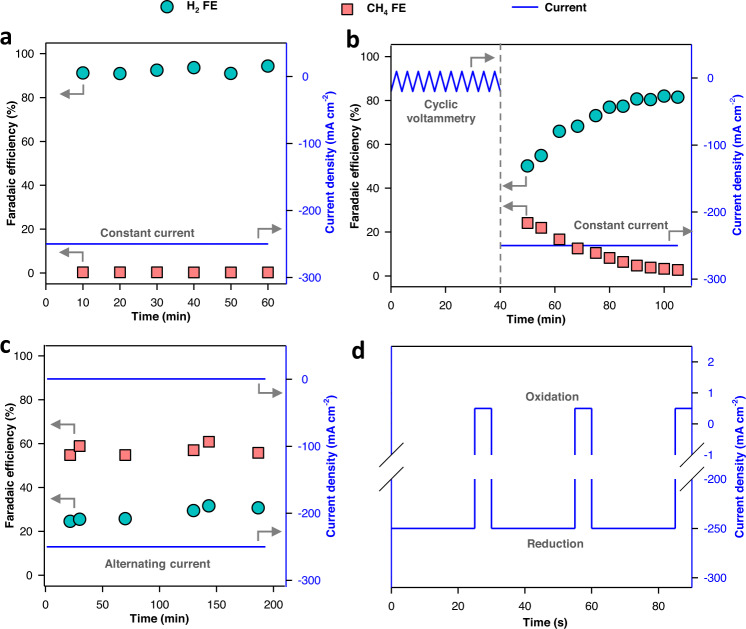
Electrocatalytic CO_2_ reduction with constant and alternating current operations. **a** Product distribution of Cu mesh over time operated at a constant reduction current density of 250 mA cm^−2^. **b** Product distribution of Cu mesh after catalyst surface activation using cyclic voltammetry (CV) between −20 and 10 mA cm^−2^ for 10 cycles, followed by operation at a constant reduction current density of 250 mA cm^−2^. **c** Product distribution of Cu mesh operated using alternating negative and positive currents (oxidation current density of 0.5 mA cm^−2^ and oxidation time of 3 s; reduction time of 25 s and CO_2_ reduction current of 250 mA cm^−2^). **d** Magnification in time of the square-wave alternating reduction and oxidation current density in (**c**).

We first performed the electrochemical reaction using as-received Cu mesh at a current density of 250 mA cm^−2^. In this configuration, H_2_ is the major product with Faradaic Efficiency (FE) over 90% throughout the test while the FE for CH_4_ is less than 0.5% (Fig. 2a).

To improve CO_2_ reduction selectivity and to suppress H_2_ evolution reaction, we sought to reconstruct the surface of Cu catalyst using an electrochemical oxidation−reduction process to form Cu oxide-derived catalysts, which have been found to be selective catalysts for CO_2_ reduction to hydrocarbons^41–45^. To form the Cu-oxide-derived catalyst, we performed repeated cyclic voltammetry (10 cycles) in the current density range of −20 to +10 mA cm^−2^ before performing CO_2_ reduction (Fig. 2b). We found that the CH_4_ FE increased to 25% while the H_2_ FE decreased to 50% when Cu surface is reconstructed via reduction–oxidation cycling (Fig. 2b). However, the CH_4_ FE rapidly decreased to 3% after 60 min of reduction time. These results suggest that the oxide-derived Cu surface can be selective but unstable for CH_4_ formation.

From the results above, we reasoned that periodic Cu surface reconstruction would enable selective and stable CH_4_ formation. To this end, we applied a square-wave alternating current in which the electrical current alternated between a negative and a positive current. Using this operation mode, the catalyst is periodically oxidized during CO_2_ reduction (Fig. 2c, d). The oxidation current density and time were fixed at 0.5 mA cm^−2^ and 3 s, respectively, and the reduction time was 25 s. We found that, under these conditions, CH_4_ was the major product with an FE of 55–60% maintained throughout the reaction. Meanwhile, H_2_ formation is suppressed, with a stable FE around 30% (Fig. 2c). Other minor products include C_2_H_4_ (1–3%) and CO (<1%). During the oxidation cycle, H_2_ produced from the reduction cycle may be oxidized, contributing to the observed low H_2_ FE. However, because the number of charges during the oxidation cycle (0.0015 C cm^−2^) is much smaller than those in the reduction reaction (6.25 C cm^-2^), this possible contribution is insignificant.

### Optimizing oxidation and reduction conditions

**Fig. 3:**
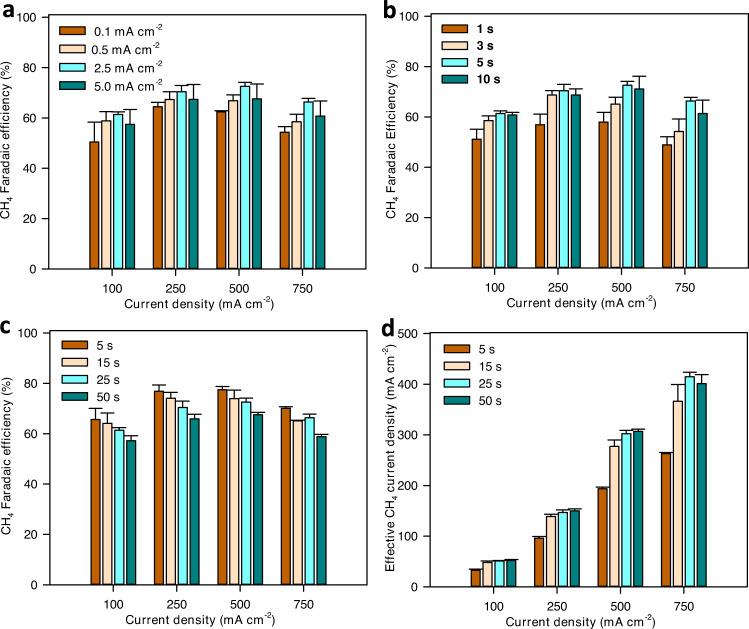
Optimization of oxidation and reduction conditions. **a** Effect of oxidation current on CH_4_ selectivity. Oxidation and reduction times were fixed as 5 s and 25 s, respectively. **b** Effect of oxidation time. The oxidation current density was fixed at 2.5 mA cm^−2^ and the reduction time was 25 s. **c** Effect of reduction time. Oxidation current density and time were fixed as 2.5 mA cm^−2^ and 5 s, respectively. **d** Effect of reduction time on effective CH_4_ partial current density. The effective CH_4_ current was calculated as follows: effective current = (total current) × (Faradaic efficiency) × (time for reduction cycle)/(time for reduction cycle + time for oxidation cycle). Data for FE of other gas products are shown in Figs. S8–S10. The error bars represent SD (*n* = 3 independent replicates).

Having identified a strategy for the selective production of CH_4_, we sought to further optimize the oxidation-induced Cu surface modification by varying the oxidation and reduction current and time. We first studied the effect of oxidation current on CH_4_ selectivity. The oxidation time was fixed at 5 s while we varied the oxidation current density between 0.1 and 5 mA cm^−2^. The reduction time was fixed at 25 s and the reduction current density varied in the range of 100–750 mA cm^−2^. We found that higher oxidation current density generally improves CH_4_ selectivity (Fig. 3a). Particularly, with an oxidation current density of 2.5 mA cm^-2^, CH_4_ FE of over 70% was achieved at 250 mA cm^−2^ and 500 mA cm^−2^. At a current density of 750 mA cm^−2^, we achieved a CH_4_ FE of 67.8%, corresponding to a partial CH_4_ current density of 508.5 mA cm^−2^. C_2_H_4_ is the other main product with an FE in the range of 2–6%. The total FE for liquid products is less than 5% (Fig. S6). The full cell voltage was 3.52 V at a current density of 100 mA cm^−2^ and increased to 4.25 V, 5.4 V, and 7.57 V as the current density increased to 250 mA cm^−2^, 500 mA cm^-2^, and 750 mA cm^−2^, respectively (Figs. S6, S7). When the oxidation current density is higher than 2.5 mA cm^−2^, further increases in oxidation current do not improve CH_4_ selectivity (Fig. 3a).

To study the effect of oxidation time, we fixed the oxidation current density at 2.5 mA cm^−2^ and varied the oxidation time in the range of 1 to 10 s. As with oxidation current, high oxidation time favors CH_4_ production, especially at high current densities (Fig. 3b). To investigate the effect of reduction time, we fixed oxidation current density and time at 2.5 mA cm^−2^ and 5 s, respectively, and varied the reduction time in the range of 5 to 50 s. Reducing the reduction time increases CH_4_ selectivity substantially (Fig. 3c). With a reduction time of 5 s, CH_4_ FE higher than 70% was achieved at all current densities in the range of 100–750 mA cm^−2^. Notably, CH_4_ FE reached 77% at 500 mA cm^−2^ and a CH_4_ partial current of 525 mA cm^−2^ was achieved at current density of 750 mA cm^−2^. We reason that highly selective Cu species are generated during the oxidation step. During the reduction cycle, the Cu surface is gradually reconstructed leading to a decrease in CH_4_ selectivity. Thus, shortening reduction time enables the efficient use of highly selective Cu species, leading to higher CH_4_ selectivity. It is also possible that as the reduction timescale becomes sufficiently short, replenished CO_2_ within the catalyst domain enables higher CH_4_ selectivity.

While decreasing reduction time increases instantaneous CH_4_ selectivity, it also reduces the effective duty cycle for CO_2_ reduction (reduction time/(reduction time + oxidation time)). To evaluate the effective production rate of CH_4_, we normalized CH_4_ partial current density to total operation time (reduction and oxidation time). The highest effective CH_4_ partial current recorded was over 400 mA cm^-2^, achieved with a reduction time of 25 s (Fig. 3d). Longer reduction times lower CH_4_ selectivity while shorter reduction times reduce effective operating time. Our results show a tradeoff between CH_4_ selectivity and effective partial current density towards reduction time.

### Catalyst surface changes induced by oxidation

**Fig. 4:**
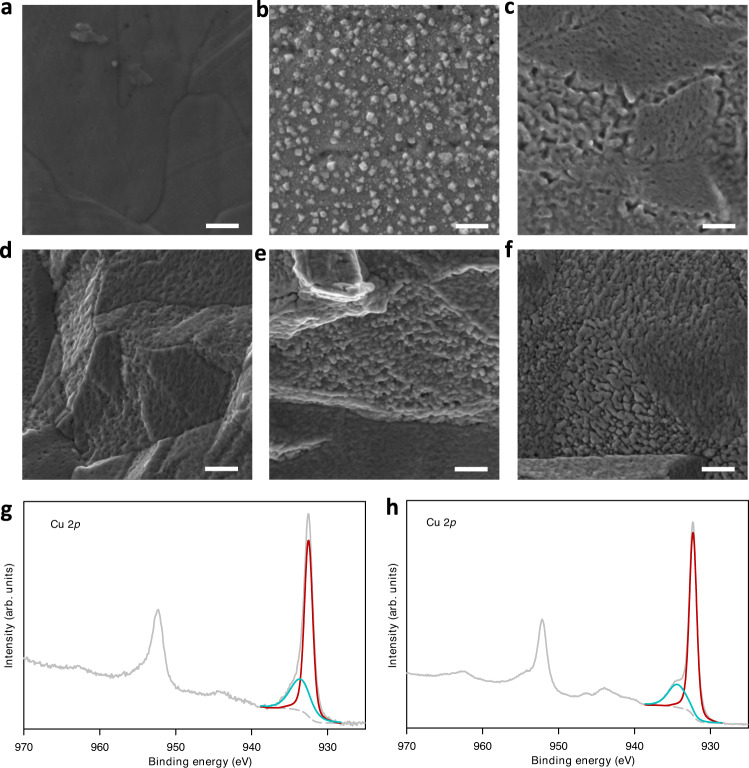
Surface changes induced by alternating reduction–oxidation current. **a,** **b** Scanning electron microscopy (SEM) images of Cu mesh electrode before (**a**) and after (**b**) CO_2_ reduction using fixed reduction current. Samples SEM image after electrolysis with alternating reduction–oxidation times of 25 s–5 s (**c**), 50 s–5 s (**d**), 15 s–5 s (**e**), and 5 s–5 s (**f**). Cu 2*p* X-ray photoelectron spectroscopy (XPS) spectra of Cu mesh electrode after CO_2_ reduction using fixed currents (**g**); and alternating 25 s–5 s reduction–oxidation current (**h**). The samples for SEM and XPS were collected after being tested at 100, 250, 500, and 750 mA cm^−2^ reduction current densities for 40 min at each current regime (total reaction time of 160 min). The oxidation current density and time were 2.5 mA cm^−2^ and 5 s, respectively. The reduction time considered for samples (**c**)–(**f**) are 25 s, 50 s, 15 s, and 5 s, respectively. Scale bar in Fig. **a**–**f** = 200 nm.

To understand the effect of different operating procedures on the surface changes of the catalysts, we performed scanning electron microscopy (SEM) characterization of the catalyst before and after CO_2_ reduction reactions. We found that CO_2_ reduction conditions have a significant impact on the surface of Cu catalysts. SEM characterizations after ECR reactions with constant reduction current reveal the formation of Cu nanoparticles with irregular shapes (Figs. 4b, S12, S13) as opposed to a smooth surface of the sample before the reaction (Figs. 4a, S11). This observation is consistent across different locations of multiple samples. Previous studies using operando characterizations of catalysts under ECR conditions suggested that the Cu surface is dynamic and undergoes significant morphological changes due to multiple processes, including dissolution/redeposition, agglomeration, fragmentation and reshaping^33,34,46–48^. Formation of Cu nanoparticles on the Cu surface during ECR has been observed in a previous study at a current density below 50 mA cm^−2^ ref. ^12^. Because the surface changes are accelerated at higher reaction rates or more negative applied potentials^33^, formation of Cu nanoparticles at current densities of 500 to 750 mA cm^−2^ in our system is reasonable. In sharp contrast to the constant reduction operation, alternating reduction–oxidation currents were found to induce the formation of a porous Cu surface (Fig. 4c). The porous structure becomes more pronounced when the reduction time is reduced from 50 s to 5 s (Figs. 4d–f, S14), suggesting that oxidation cycle frequency is an important factor governing the morphology of the catalyst.

To study the changes in the surface composition of the catalysts induced by the different operating protocols, we performed X-ray photoelectron spectroscopy (XPS) analyses of the samples before and after CO_2_ reduction reactions (Figs. 4g, h, S15). All samples show the presence of both metallic copper (Cu), characterized by the peak at a binding energy of 932 eV, and copper oxide (CuO), indicated by the peak at a binding energy of 933.5 eV (Fig. 4g, h). The ratio of the two deconvoluted peaks at 932 and 933.5 eV are similar between the two samples, indicating a similar ratio of copper oxide and metallic copper. The presence of copper oxide on the Cu electrodes after the reaction are likely due to Cu oxidation during exposure to air.

### Origin of high CH_4_ selectivity

**Fig. 5:**
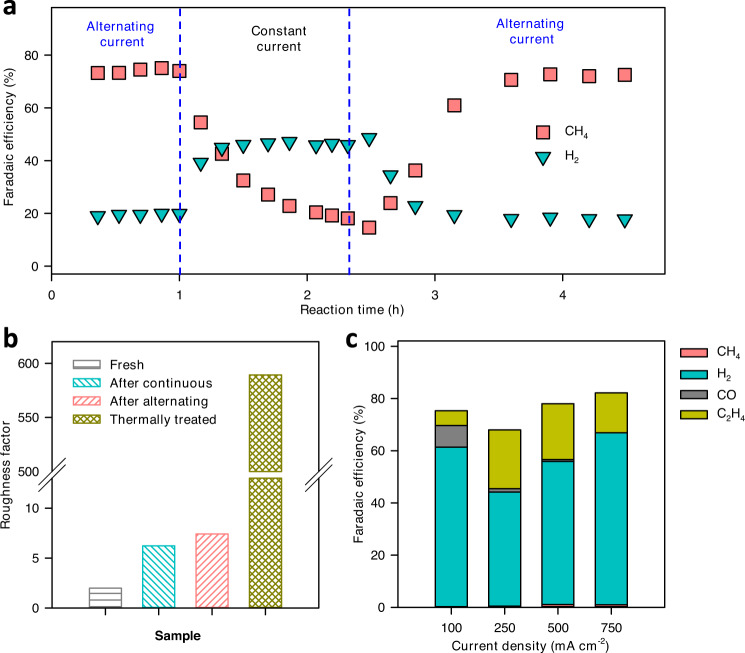
Roles of operation mode and surface roughness factor. **a** Product distribution of Cu mesh over time operated using alternating currents (oxidation current density of 2.5 mA cm^−2^ and oxidation time of 5 s; reduction time of 25 s and CO_2_ reduction current of 250 mA cm^−2^), followed by constant reduction current at 250 mA cm^−2^ and then alternating currents. **b** Roughness factors of fresh Cu mesh, Cu mesh after constant reduction current operation, Cu mesh after alternating current operation, and thermally treated Cu mesh after alternating current operation. **c** Product distribution of thermally treated Cu mesh using alternating current operation. Samples after reaction were collected after being tested at 100, 250, 500, and 750 mA cm^−2^ current densities for 40 min at each current density (total reaction time of 160 min). For samples with alternating current operations, the oxidation current density was 2.5 mA cm^−2^, the oxidation and reduction times were 5 s and 25 s, respectively.

Previous studies of ECR operating at low current densities of 10–50 mA cm^−2^ in aqueous systems have suggested three key factors that affect hydrocarbon selectivity, including the nature of Cu active sites^44,49,50^, catalyst surface roughness or loading^51–53^, and the electrolyte^54^. In our system, we found that catalyst morphology and alternating current operation are two crucial factors for high CH_4_ production. To understand the effect of each factor, we performed a series of control experiments to decouple their roles. To investigate the contribution of operating mode, we performed ECR at a current density of 250 mA cm^−2^ using alternating current until a stable CH_4_ FE of over 70% was achieved for 1 h (Fig. 5a) and then switched the reaction to the constant current mode at the same current density. The CH_4_ FE drops quickly to 20% after 1 h of reaction while H_2_ FE increases significantly. When the system was switched back to alternating operating mode again, CH_4_ FE recovered to the initial FE of over 70%. These results confirm that even with oxidation-induced activated Cu surface, alternating current operation is needed to maintain high CH_4_ FE in aqueous fed system at high current densities.

To study the possible effect of impurities such as Ni and Fe ions, which could leach from the Ni foam anode and be transported to the cathode during alternating current operation^55^, we performed ECR using stable IrO_x_ supported on titanium felt as the anode. This yielded similar CH_4_ FEs compared to Ni-based anodes (Fig. S16). We also intentionally added Ni and Fe ions to the catholyte with concentrations ranging from 0.1 to 1 ppm and found that Ni and Fe impurities both show detrimental effects on CH_4_ production (Figs. S17, S18). These results confirm that potential Ni and Fe impurities do not contribute to the high CH_4_ FE obtained in our systems.

To study the effect of surface morphology, we estimated the surface roughness factors of the catalysts from double-layer capacitance measurements. Cu electrodes after both constant and alternating current operations show similar roughness factors of 6-8 which is ca. 3 times higher than that of fresh Cu mesh (Figs. 5b, S19). We reason that a relatively low surface roughness factor is the main reason for the high CH_4_ FE observed in our study. This is consistent with what has been demonstrated in aqueous ECR systems using Cu mesh and Cu foils as electrodes, suggesting that a balance between surface roughness and applied potential is crucial for high CH_4_ selectivity^53^. To further confirm the effect of surface roughness, we prepared a high surface area Cu mesh electrode using a thermal treatment process^56^. With a high surface roughness factor of 590, the electrode produced C_2_H_4_ as the main hydrocarbon product (FE of 15–25%) while CH_4_ production is suppressed (FE less than 1%) in current densities of 250 to 750 mA cm^-2^ using alternating current operation (Figs. 5c, S20). These results suggest that both low roughness factors and alternating current operation are needed to achieve high CH_4_ FE and production rates in our aqueous solution ECR system.

Finally, the local pH in the catalyst layer, estimated from numerical simulation for the 0.3 M KHCO_3_ catholyte, is between 10 and 12 at current densities of 100–500 mA cm^-2^. This suggests that water proton source for CH_4_ formation (Fig. S21). Our results are consistent with previous works showing an optimal local pH of 10.5–11 for CH_4_ formation on a relatively flat Cu surface^53^. Experiments using lower KHCO_3_ catholyte concentration show a higher C_2_H_4_:CH_4_ ratio (Fig. S22), further supporting the role of pH within the catalyst layer to tune hydrocarbon selectivity. Lower KHCO_3_ concentrations have reduced buffering effects, leading to higher local pH near the flow channel (Fig. S21), presumably outside of the optimal pH range for CH_4_ formation.

### Experimental validation of CO_2_ sources

As discussed above, there are two sources of CO_2_ in the aqueous solution-fed system: CO_2_ dissolved in the electrolyte and CO_2_ generated in-situ from protons reacting with bicarbonate. To understand the role of each source, we performed a series of controlled experiments. First, we performed ECR using CO_2_ saturated KHCO_3_ electrolyte and an anion exchange membrane (AEM). In this configuration, dissolved CO_2_ is the only CO_2_ source because the in-situ pathway is blocked due to lack of protons. We found that dissolved CO_2_ enables high CH_4_ selectivity (up to 52%) at low current densities of 100 mA cm^−2^ and 250 mA cm^−2^ (Fig. S23). At the higher current densities of 500 mA cm^−2^ and 750 mA cm^−2^, CH_4_ FE is decreased to below 40%, which is much lower than those obtained using CO_2_-saturated KHCO_3_ and a BPM (Fig. S23). These results suggest that dissolved CO_2_ can serve as the main CO_2_ source for CH_4_ production at low current densities (below 250 mA cm^−2^) but is not sufficient to maintain high CH_4_ selectivity at higher current densities (above 500 mA cm^−2^).

To explore the role of in-situ generated CO_2_ gas, we performed the reaction using an N_2_ saturated KHCO_3_ electrolyte and a BPM. In this configuration, the contribution of dissolved CO_2_ is limited to the equilibrium dissolved CO_2_ concentration of the electrolyte (~1.5 mM vs. 33 mM in a CO_2_-saturated case^57^). While in-situ generated CO_2_ enables the formation of CH_4_, its FE is relatively low. The highest recorded CH_4_ FE was 27%, achieved at 500 mA cm^−2^, corresponding to a CH_4_ partial current density of 135 mA cm^−2^ (Fig. S23), which is comparable to previous reported data using in-situ generated CO_2_ gas from bicarbonate electrolyte^27^. These results confirm that the presence of dissolved CO_2_ is crucial for high CH_4_ FE and both CO_2_ sources are required for high CH_4_ selectivity at high current densities.

We also studied the effect of catholyte KHCO_3_ concentration on ECR performance. KHCO_3_ concentration can influence CO_2_ solubility, the amount of in-situ CO_2_ generated, and the local pH on the catalyst surface. Electrolyte with low KHCO_3_ concentration has higher CO_2_ solubility and yields higher local pH on the catalyst surface during ECR due to reduced buffering. Meanwhile, high KHCO_3_ concentration increases the availability of the HCO_3_^−^ ion at the membrane surface for in-situ CO_2_ generation. At the current density of 100 mA cm^−2^, the CH_4_ FEs with 0.1 M and 0.3 M KHCO_3_ electrolyte are much higher than that of 1 M KHCO_3_, suggesting the important role of high local pH at low current densities (Fig. S22). At high current densities (500–750 mA cm^−2^), 0.3 M KHCO_3_ yields a substantially higher CH_4_ FE compared to either 0.1 M or 1 M KHCO_3_ (Fig. S22). These results suggest that a balance between dissolved CO_2_, in-situ generated CO_2_, and local pH is needed to achieve high CO_2_-to-CH_4_ conversion. Compared to the dense electrodes used in previous studies^27^, the open matrix catalyst used in this work effectively utilizes dissolved CO_2_ as the carbon source. Therefore, highly concentrated KHCO_3_ electrolyte is not needed to provide enough in-situ generated CO_2_. As a result, the open matrix electrode allows the utilization of KHCO_3_ electrolyte with relatively low concentration, which produces favorable local pH for CO_2_ conversion.

To validate the experimental trends and the conclusions from the multiphysics model, the model was tested against various experimental configurations, including AEM operation (Fig. S24), various sparging gases (Fig. S24), and at different electrolyte concentrations (Fig. S25). The model can replicate cell performance trends in all cases.

### Product concentration and system stability

**Fig. 6:**
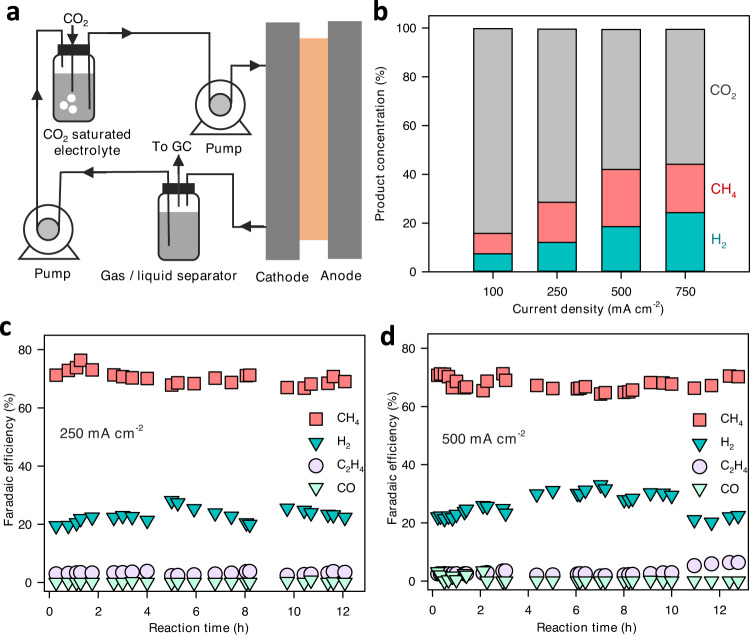
Product concentration and system stability. **a** Schematic illustration of the experimental setup for analyzing product concentration. **b** H_2_, CH_4_, and CO_2_ concentrations in the outlet gas stream at different current densities. Gas product distribution over time at current densities of 250 mA cm^−2^ (**c**) and 500 mA cm^−2^ (**d**) shows the stability of the system. The operation conditions in Fig. (**b**)–(**d**) were as follows: 25 s reduction time, 2.5 mA cm^−2^ oxidation current density, and 5 s oxidation time.

To analyze product concentration in the outlet stream, we designed a system in which the gas product is separated from CO_2_ bubbling solution. Gas and liquid electrolyte go through a separator. Gas products were collected for analysis while liquid electrolyte is recycled to the CO_2-_saturating solution (Fig. 6a). CH_4_, H_2_, and CO_2_ are the main components in the gas products. At 100 mA cm^−2^, both CH_4_ and H_2_ concentrations are relatively low, while CO_2_ makes up the bulk of the gas stream (Fig. 6b). At higher current densities, CH_4_ and H_2_ concentration increases, reaching a maximum concentration of 23.5% for CH_4_ at 500 mA cm^−2^. The molar ratio of CH_4_ produced to unreacted CO_2_ gas was 40.7%, exceeding the highest previously reported of 34%^27^. During the reaction, CO_2_ gas is produced from the reaction of bicarbonate and protons from the BPM (Eq. 1). At the same time, CO_2_ is consumed via the formation of CH_4_ (Eq. 2) and the reaction with hydroxyl ions (OH^−^) during the reaction.

3 $${{{{{{\rm{CO}}}}}}}_{2}+{{{{{{\rm{OH}}}}}}}^{-}\to {{{{{{{\rm{HCO}}}}}}}_{3}}^{-}$$

In principle, all CO_2_ produced from Eq. (1) can be converted to CH_4_ or HCO_3_^−^ via Eqs. (2) and (3) because the amount of H^+^ and OH^−^ produced are equal. Excess amount of CO_2_ observed in the gas stream could be due to the low efficiency of the reaction (3) or slow absorption from gas phase CO_2_ bubbles into the bulk electrolyte. We reason that a well-designed gas/liquid separator that allows efficient CO_2_ conversion to bicarbonate would significantly reduce the amount of CO_2_ gas in the product stream.

To evaluate the stability of the system, we performed the reaction at current densities of 250 and 500 mA cm^−2^ and tracked the gas products over time (Figs. 6c, d, S26). At 250 mA cm^−2^, a CH_4_ FE in the range of 70–75% was maintained over a period of 12 h. H_2_ and CO FEs were relatively constant while ethylene FE increased slightly from 2% to 4% after 12 h of reaction. A similar trend was observed at the current density of 500 mA cm^−2^ with CH_4_ FE being stable at around 65 - 70% throughout the experiment of 12 h in duration (Fig. 6d).

## Discussion

**Fig. 7:**
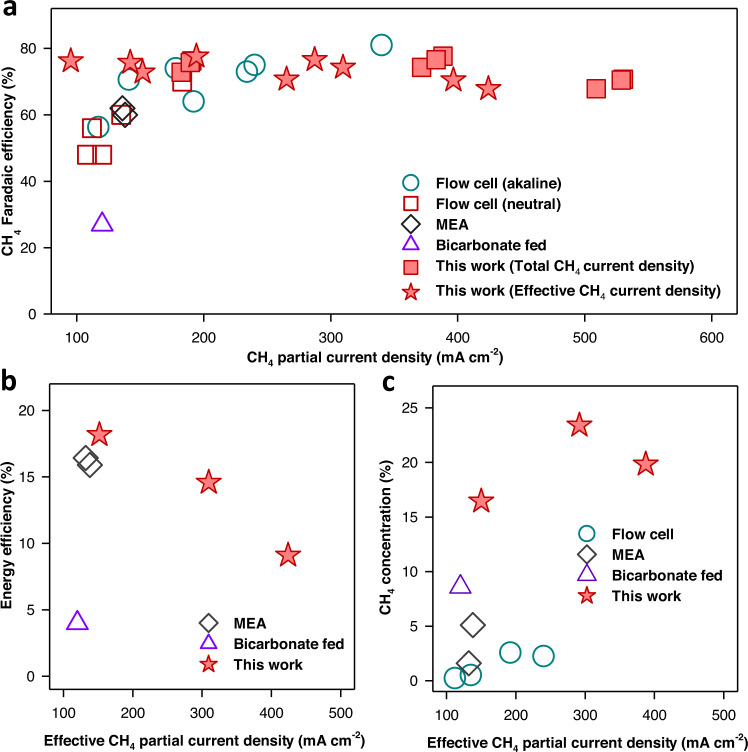
Performance comparison between ECR systems. Comparison of our work with previous studies on CO_2_-to-CH_4_ conversion with partial current densities over 100 mA cm^−2^ using a flow cell (with alkaline electrolyte^11–17^ and pH-neutral electrolyte^18–22^), an MEA cell with AEM^2, 11^, and bicarbonate fed using BPM^27^. **a** CH_4_ Faradaic efficiency. **b** Energy efficiency. **c** CH_4_ product concentration at the outlet stream. Effective CH_4_ partial current density was used for Fig. 7b, c. Detailed data are shown in Table S1.

High CH_4_ selectivity at relatively high partial current density can be obtained using an alkaline flow cell system (Fig. 7a and Table S1)^11–17^. However, carbonate formation in this system requires a significant amount of energy for electrolyte regeneration. Neutral-pH electrolytes can reduce carbonate formation but often lead to lower CH_4_ selectivity at relatively lower partial current density compared to alkaline flow cells (Fig. 7a and Table S1)^18–22^. While high CH_4_ selectivity has been achieved with flow cells, the flow cell platform typically exhibits a large cell resistance, requiring high cell voltages to achieve high current densities. Thus, energy efficiency has not been reported for flow cell systems^11–22^. MEA cells using an anion exchange membrane can produce CH_4_ at relatively high selectivity and energy efficiency (Fig. 7a, b)^2^. However, CO_2_ crossover with anion exchange membranes requires additional CO_2_ separation. In both flow cell and MEA systems, CH_4_ produced is diluted with the unreacted CO_2_ gas stream leading to low CH_4_ product concentration (Fig. 7c). Previous bicarbonate-fed systems produced CH_4_ with relatively high concentration and minimized CO_2_ crossover. However, they exhibit low energy efficiency, FE, and partial current density compared to those obtained with flow cell and MEA systems (Fig. 7)^27^.

We developed a bicarbonate-fed system which achieves the high CH_4_ selectivity (>70%) typically found in flow cell configurations using gas-phase CO_2_ electrolysis. The obtained CH_4_ partial current density of over 400 mA cm^−2^ exceeds most of what has been previously achieved in both flow cell and MEA systems (Fig. 7a). Our system delivers an energy efficiency of 15–18% in the current range of 250–500 mA cm^−2^ which are comparable to previous MEA systems while minimizing carbonate crossover (Fig. 7b). Particularly, our dissolved CO_2_ and bicarbonate-fed system also delivers the highest CH_4_ product concentration compared to all previous CO_2_-to-CH_4_ systems (Fig. 7c). The performance of the architecture is facilitated by in-situ surface activation of the copper catalyst combined with facile liquid transport enabled by an open matrix structure. An optimized surface activation strategy is presented which balances surface reconstruction with maximizing effective current density. The large pore structure of the catalyst allows both dissolved CO_2_ and CO_2_ generated in situ to be efficiently utilized, as confirmed by both experiment and multiphysics modeling. Further improvements to the system performance are possible through the suppression of CO_2_ bubbling and optimization of the gas/liquid separation scheme.

## Methods

### Electrochemical CO_2_ reduction

Electrochemical measurements were performed in a two-electrode MEA flow cell. Details of the electrolysis cell and the electrolysis system employed in this work are described in Fig. S5. In the MEA flow cell, copper mesh (100 pores per inch (ppi)) and nickel foam (1.5 mm thickness; 80–100 ppi, MTI Corp.) were used as the cathode and anode, respectively, and were separated by a bipolar membrane (Fumasep) or an anion exchange membrane (Fumasep). The exposed sizes of both the cathode and anode electrodes were 1 cm × 2 cm (a geometric area of 2 cm^2^ was used for all current density calculations). The copper mesh was configured to be in direct contact with the membrane. The polytetrafluoroethylene (PTFE) gasket was used to prevent contact between anode and cathode titanium flow plates. A PTFE spacer was used to avoid direct contact between the Cu mesh and the cathode flow plate.

For the electrolysis system, a potentiostat (Autolab PGSTAT204) with a current booster (Metrohm Autolab, 10 A) was used for all experiments. Two peristaltic pumps were used to circulate the anolyte (1 M KOH; 200 mL) and catholyte (0.1 M, 0.3 M, or 1 M KHCO_3_; 1000 mL) between the reservoirs and the electrochemical cell at a flow rate of 30 mL min^−1^. Before the measurement, CO_2_ gas (Praxair, 99.99%) or N_2_ gas (Praxair, 99.99%) was purged in the KHCO_3_ aqueous solution for at least 30 min. CO_2_ was continuously purged into the catholyte throughout the experimental process (during both oxidation and reduction cycles) at a flow rate of 50 standard cubic centimeters per minute (sccm). Electrolysis was carried out for 2500 s at each tested current density, with the reaction gaseous product being analyzed every 15 min. Gas products coming out from the cell were carried by the CO_2_ or N_2_ stream bubbled through the catholyte reservoir to a 6-way valve where it was injected to a gas chromatography (GC, PerkinElmer Clarus 590) for quantification. The GC is equipped with a thermal conductivity detector (TCD) operated at 180 °C and a flame ionization detector (FID) operated at 250 °C. A molecular sieve (5A) packed column (Supelco) connected to the TCD was used to analyze CO, CO_2_, and H_2_ products while a Carboxen-1000 packed column (Supelco) connected to the FID was employed to quantify CH_4_, C_2_H_4_ and other potential hydrocarbons. Both columns were operated at a fixed temperature of 180 °C and with Ar carrier gas (flowrate of 20 sccm).

Aliquots of the liquid products were analyzed using nuclear magnetic resonance spectroscopy (NMR)^1^.H NMR spectra of freshly collected liquid products were acquired on an Auto-400 ultrashield Bruker Instrument operating at denoted spectrometer frequency given in megahertz (MHz) at 25 °C in D_2_O using water suppression mode, with dimethyl sulfoxide as the internal standard.

The FE of ECR gas products was calculated as follows:

$${{FE}}_{k}=\frac{{n}_{k}\cdot F\cdot {x}_{k}\cdot {F}_{m}}{I}\times 100\%$$

Where $F{E}_{k}$ is the FE of product ${k\ }$, ${n}_{k}$ is the number of electrons (for $k$= CH_4_, ${n}_{k}$ is 8) transferred to form gaseous product ${k\ }$, *F* is Faraday’s constant (96,485 C mol^−1^), ${F}_{m}$ is the molar flow rate of the gas outlet stream in $\frac{{mol}}{s}$. ${x}_{k}$ is the molar fraction of the gas product k in the gas outlet stream during reduction cycle. ${I\ }$ is the total (applied) current in Amperes (${A\ }$) during reduction cycle.

Because the reduction and oxidation cycles are relatively short and the CO_2_ is constantly flowing through the system (during both reduction and oxidation cycles), gas produced during the reduction cycle is diluted by CO_2_ flow during the oxidation cycle. Thus, the actual molar fraction of the gas product during the reduction cycle is calculated as follows:

$${x}_{k}={{x}^{{\prime} }}_{k}\cdot \frac{\triangle {t}_{r}+\, \triangle {t}_{o}}{\triangle {t}_{r}}$$

where ${{x{{\hbox{'}}}}}_{k}$ is the diluted concentration (mixing of gas production in both oxidation and reduction cycle) measured by the Gas chromatography. $\triangle {t}_{r}$ and $\triangle {t}_{o}$ are the reduction and oxidation time in each reduction–oxidation cycle.

The FE of ECR liquid products were calculated as follows:

$$F{E}_{l}=\frac{n\cdot F\cdot \triangle {\delta }_{l}}{\triangle Q}\times 100\%$$

Where $n$ is the number of electrons transferred to form liquid product $l$, *F* is the Faraday’s constant (same as above), Δ*δ*_*l*_ is the accumulated number of moles of the corresponding product $l$. $\triangle Q$  is the total charge transfer during the electrolysis.

CH_4_ partial current density was calculated in similar way as:

$${j}_{CH_{4}}=\frac{I\cdot F{E}_{C{H}_{4}}}{a}$$

Where ${j}_{C{H}_{4}}$ is the partial current density of CH_4_. ${a\ }$ is the geometric surface area (${a\ }$ = 2 cm^2^ in our system). *I* is the total current during the reduction cycle.

Effective CH_4_ current density was calculated as follows:

$${J}_{C{H}_{4},{eff}}=I\cdot F{E}_{C{H}_{4}}\cdot \left(\frac{\triangle {t}_{r}}{\triangle {t}_{r}+\triangle {t}_{o}}\right)$$

where ${J}_{C{H}_{4},{eff}}$ is the effective CH_4_ current density. The effective current density reflects the effective operation time of the electrolyzer considering both the time for CO_2_ conversion (reduction) and the time for catalyst regeneration (oxidation). $\triangle {t}_{r}$ and $\triangle {t}_{o}$ are the reduction and oxidation times in each reduction–oxidation cycle. The factor $\frac{\triangle {t}_{r}}{\triangle {t}_{r}+\triangle {t}_{o}}$ is used in the expression to weight operation (conversion) time to the total time.

The energy efficiency, EE, was calculated as follows:

$${EE}=\left(\frac{{E}^{o}\cdot F{E}_{C{H}_{4}}}{{V}_{{cell}}}\right)$$

Where ${V}_{{cell}}$ is the applied cell potential for a given geometric current density. ${E}^{o}$ = 1.06 is the equilibrium potential for the reaction: CO_2_ + 2H_2_O = CH_4_ + 2O_2_.

### IrO_x_/Ti felt preparation

The IrO_x_/Ti felt was prepared using a drop-casting method. A mixture of 40 mg of IrO_x_ (Fuel Cell Store) and 160 µL of 5 wt% Nafion perfluorinated resin solution (Sigma-Aldrich) in 5 ml methanol was sonicated for 30 min. Then 1 mL of the resulting mixed solution was drop-casted onto a Ti felt substrate (1 cm × 2 cm; Fuel Cell Store). The sample was allowed to dry overnight in ambient conditions.

### High surface area Cu electrode preparation

The high surface area Cu electrode was prepared by thermal treatment. The Cu mesh was kept in the furnace and heated to 500 °C at a ramp rate of 5 °C min^−1^. The furnace temperature was maintained at 500 °C for 3 h before it was cooled down to room temperature. The resulting high surface area Cu mesh was pretreated and electro-reduced at a current density of 20 mA cm^−2^ for 300 s in H-cell with a 0.3 M KHCO_3_ catholyte solution before performing an alternating reduction/oxidation reaction.

### Electrochemical double-layer capacitance measurement

Electrochemical double-layer capacitance was determined by measuring cyclic voltammetry (CV) in an H-cell, made up of two different chambers and separated by an anion exchange membrane. A three-electrode setup was used, comprised of the working electrode and a KCl saturated reference electrode (Ag/AgCl electrode) in the cathode chamber and a platinum counter electrode placed in the anode chamber. All potentials for this measurement were determined against the Ag/AgCl reference electrode. The dimension of the working electrode which was immersed in the electrolyte is 1 cm × 1 cm. First, the potential range of the non-Faradaic current regime was determined from CV. CV measurements were then conducted in a CO_2_ saturated 0.3 M KHCO_3_ electrolyte solution by sweeping the potential in the non-faradaic region between −0.55 and −0.65 V vs. Ag/AgCl with 3 cycles for each scan rate of 0.1, 0.2, 0.4, 0.6 0.8, 1, 1.2, and 1.4 V s^−1^. For the thermally treated Cu mesh, scan rates of 0.005, 0.01, 0.02, 0.04, and 0.06 V s^-1^ were used. Roughness factors of Cu electrodes were estimated by comparing their capacitances against that of an ideally smooth Cu surface (0.029 mF cm^−2^)^56^.

### Characterizations

Catalyst surface morphology was characterized by a ZEISS Auriga FE-SEM operated at 3 kV. XPS measurements were performed using a Thermo Scientific K-Alpha spectrophotometer with a monochromated Al Kα X-ray radiation source.

### Multiphysics modeling

A one-dimensional multiphysics model was developed to describe the catalyst layer and cation exchange membrane transport dynamics. The transport and reaction of aqueous HCO_3_^–^, K^+^, CO_3_^2–^, OH^–^, H^+^, and CO_2_ are considered in the model, treated with dilute solution theory. The transport of species is governed by the Nernst-Planck equation, with the convection term neglected and electroneutrality imposed. The effective diffusion coefficient is determined with the Bruggeman correction. The porosity of the open matrix catalyst is assumed to be 0.99, based on the free transport expected in a mesoscale copper mesh, and the dense matrix 0.8^38^), matching previous works, with the gaseous fraction set to 0.2 in both cases^38^. The current distribution is determined using Ohm’s law. The electrochemical kinetics are described by the concentration-dependent Butler–Volmer equation^57^. Only the H_2_ and the CH_4_ evolution reactions are considered due to the low experimentally recorded rates of other competing reactions. The electrochemical rates are fit to the experimental performance of the copper mesh catalyst under alternating current (5 s oxidation, 25 s reduction) for CO_2_ sparging and N_2_ sparging cases. The model performance was tested against the CO_2_ sparging with an AEM case (Fig. S24). We assume that the dense matrix and open matrix catalysts share the same electrochemical rates because it is not expected that macroscale porosity would affect catalyst morphology and performance^38^. The electrode-specific surface area is assumed to be 10^4^ m^–1^ for open matrix catalyst and 10^5^ m^–1^ for the dense matrix to account for reduced specific surface area when comparing a copper mesh to a copper foam. The Donnan equilibrium boundary condition was used to describe the charge discontinuity between the catalyst layer and the cation exchange layer (CEL). The CEL has a fixed space charge density of –1.75 C m^−3^ ref. ^58^. The water hydration (mol H_2_O per mol SO_3_^–^) in the CEL was determined to be 6^59^, which defines CEL diffusivity, as in ref. ^60^ Finite-rate carbonate buffer kinetics were included throughout the catalyst and CEL domains to model HCO_3_^–^, CO_3_^2–^, OH^–^, H^+^_,_ and CO_2_ equilibrium^37^. Henry’s law was used to model CO_2_ phase transfer from liquid to vapor phase, and it was assumed that any CO_2_ bubbled away could not re-dissolve into the solution due to the fast timescales associated with bubble advection^38,39^.

The boundary condition at the end of the catalyst layer corresponds to the interface between the catalyst layer and flow plate. A mass flux boundary condition corresponding to finite mass flux from a channel with a constant Sherwood number was imposed. An assumed Sherwood number of 36.6 was used for the open matrix case and 3.66 (corresponding to laminar channel flow) for the dense matrix case to account for unsteady convection increasing catholyte flux in the open matrix case. The catholyte used was 0.1 M, 0.3 M, or 1.0 M KHCO_3_ where indicated. CO_2_ sparging was imposed by setting the CO_2_ concentration to the CO_2_ saturation concentration (33 mM), and N_2_ sparging by setting the CO_2_ concentration to the equilibrium concentration (1.5 mM for 0.3 M KHCO_3_)^59^. All other species concentrations are set to their equilibrium values, shown in Table S2. The solid-phase electric potential on the flow-plate interface is set to the half-cell potential (versus SHE), varied from 0 V to −2.1 V. The boundary condition on the other side of the domain corresponds to the interface between the cation exchange layer and anion exchange layer in a BPM. To account for the proton flux from the BPM, a boundary flux of protons equal to the integrated current density divided by the Faraday constant times the transference number is imposed as in ref. ^38^ The net ionic current is from K^+^ across the membrane. All other species (HCO_3_^–^, CO_3_^2–^, OH^–^, and CO_2_) have a no-flux condition at the CEL/AEL boundary. A transference number of 0.75 is used for the BPM cases (somewhat lower than in Lees et al. due to the comparatively lower catholyte K^+^ concentration compared to the anolyte), and 0 for the AEM case. The electrolyte potential at the CEL/AEL boundary is set to 0 V.

The model is implemented in COMSOL Multiphysics version 6.0 and solved, assuming steady state, using the PARDISO solver. The 1D domain is discretized into elements of maximum size 0.5 µm, with element sizes of 0.02 µm close to boundaries. The results were found to be independent of further mesh refinement (Fig. S27). Additional model parameters and definitions are included in Table S2 of the SI.

## Supplementary information

Supplementary Information

Peer review file

## Data Availability

All the data supporting the findings of this study are available within the article and its Supplementary Information and Source Data file. Source data are provided in this paper.

## Source data

Source Data
