# Supplementary material for: High-rate and selective conversion of CO2 from aqueous solutions to hydrocarbons
Source: Nat Commun. 2023 Jun 1;14:3176. doi: 10.1038/s41467-023-38963-y (PMC10235047; doi:10.1038/s41467-023-38963-y)
Supplement: Supplementary file 1 — Supplementary Information [file 41467_2023_38963_MOESM1_ESM.pdf]

Supplementary Information

High-Rate and Selective Conversion of CO_2 from Aqueous Solutions to Hydrocarbons

Cornelius A. Obasanjo,[1,3] Guorui Gao,[1,3] Jackson Crane,[1,3] Viktoria Golovanova,[2] F. Pelayo García de Arquer,[2] Cao-Thang Dinh[1,]*

[1]*Department of Chemical Engineering, Queen's University, Kingston, ON, K7L 3N6, Canada.*

[2]*ICFO–Institut de Ciències Fotòniques, The Barcelona Institute of Science and Technology, Barcelona, 08860, Spain.*

[3]*These authors contributed equally.*

[*]*Corresponding email: caothang.dinh@queensu.ca*

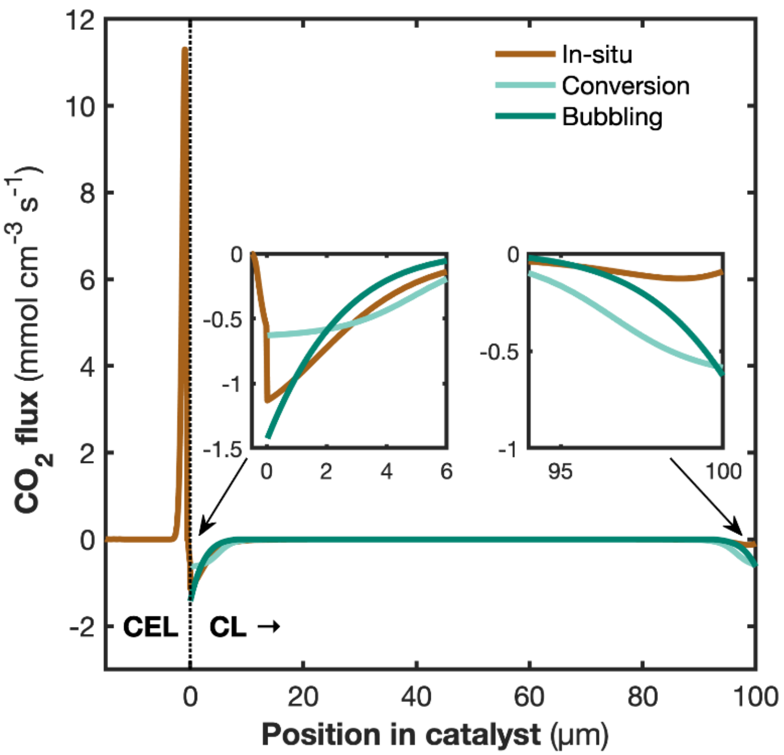

Fig. S1 | CO_2 fluxes within catalyst domain. Simulated CO_2 fluxes broken out by components (in-situ generation, CO_2 to CH_4 conversion, and bubbling from CO_2 phase transfer) as a function of position in open-matrix catalyst for 0.3 M $KHCO_3$ with CO_2 sparging at 750 mA cm^{-2}.

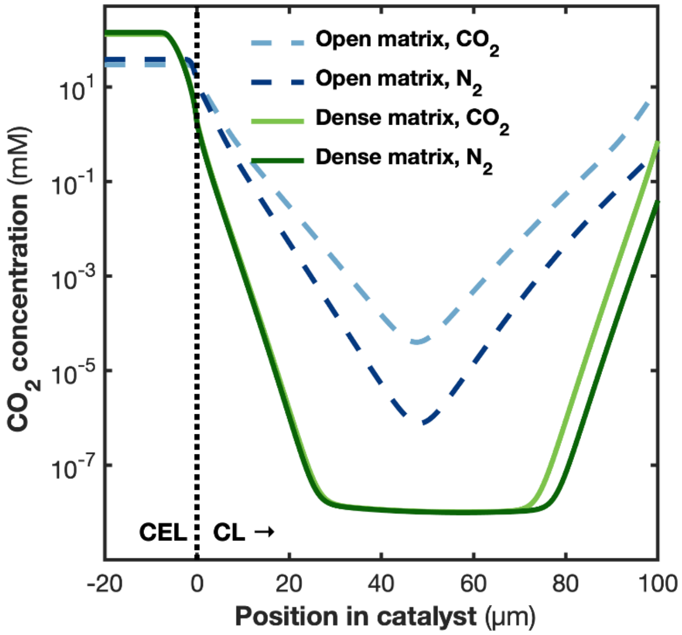

Fig. S2 | CO_2 concentration within catalyst domain. Simulated CO_2 concentration within catalyst domain for 0.3 M $KHCO_3$ at 500 mA cm^{-2} current density for dense and open matrix catalysts with CO_2 and N_2 sparging.

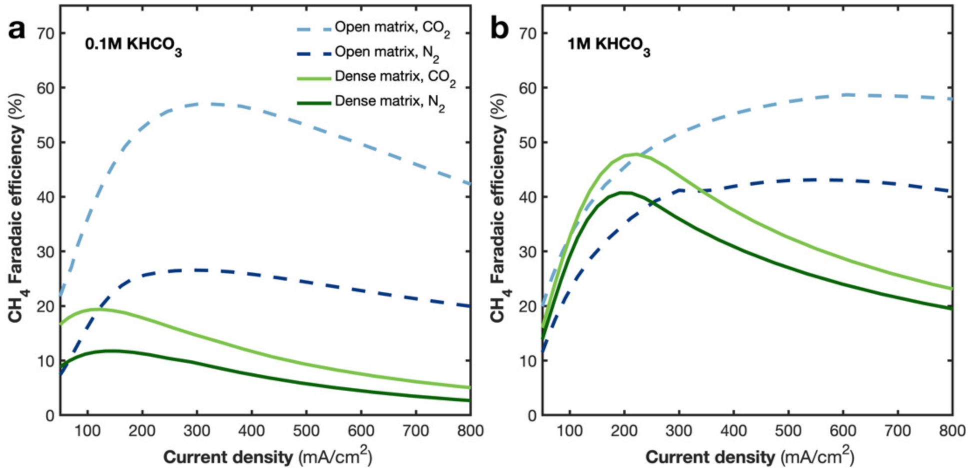

Fig. S3 | Modeled Effect of electrolyte concentration. Simulated CH_4 FE as a function of current density for (**a**) 0.1 M $KHCO_3$ and (**b**) 1 M $KHCO_3$ for dense matrix (solid lines) and open matrix (dashed lines) catalysts with CO_2 and N_2 sparging.

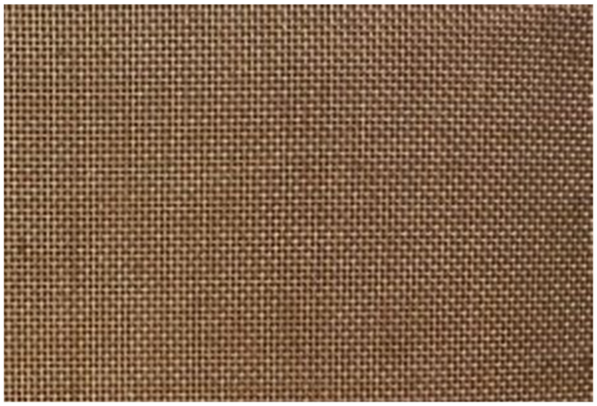

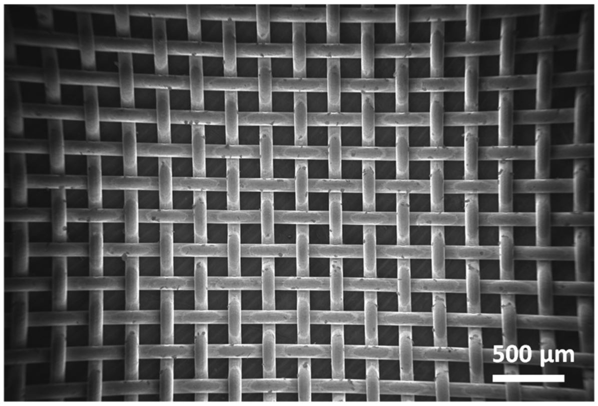

Fig. S4 | Photograph (left) and low magnification SEM image (right) of the Cu mesh used in this study.

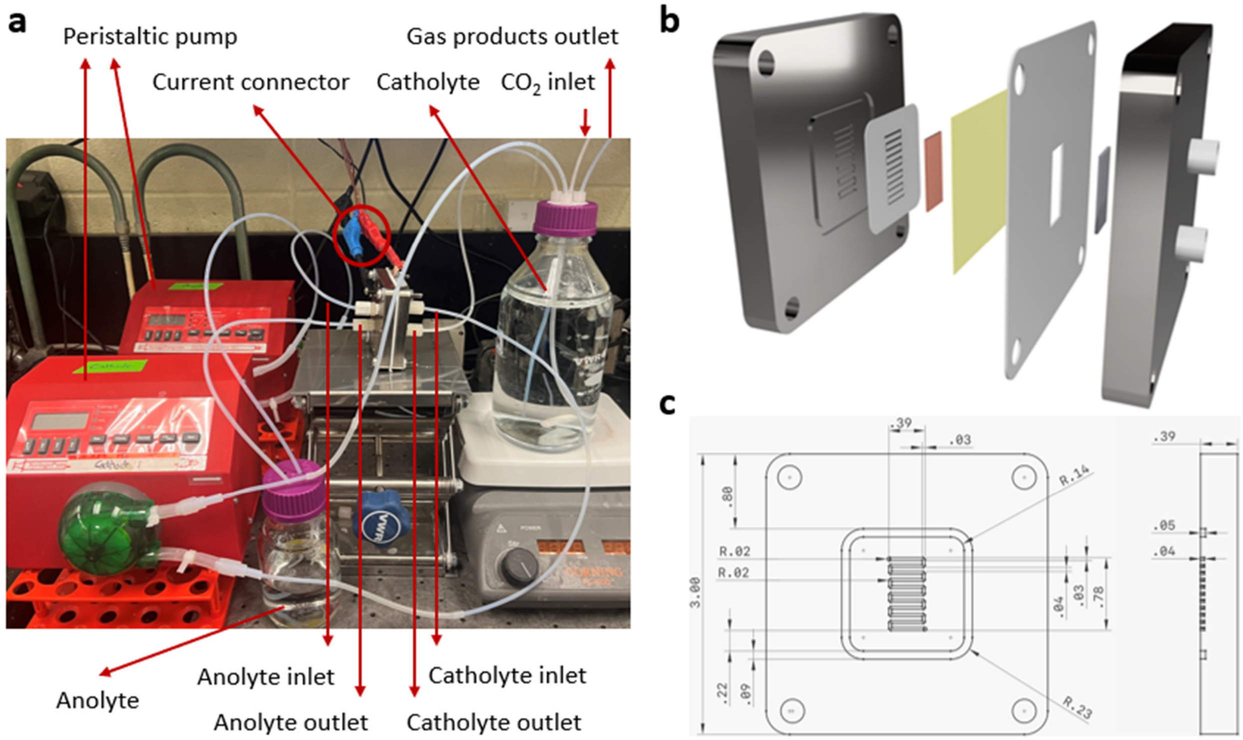

Fig. S5 | Electrochemical testing system and detailed flow channel design. (**a**) System set-up of electrochemical CO_2 conversion from aqueous solutions to hydrocarbons. (**b**) Schematic illustration of the electrolysis cell. From left to right: Cathode flow plate, PTFE spacer, copper mesh, ion exchange membrane, PTFE gasket, nickel foam, anode flow plate. (**c**) The cell dimensional drawing. All dimensions are in inches.

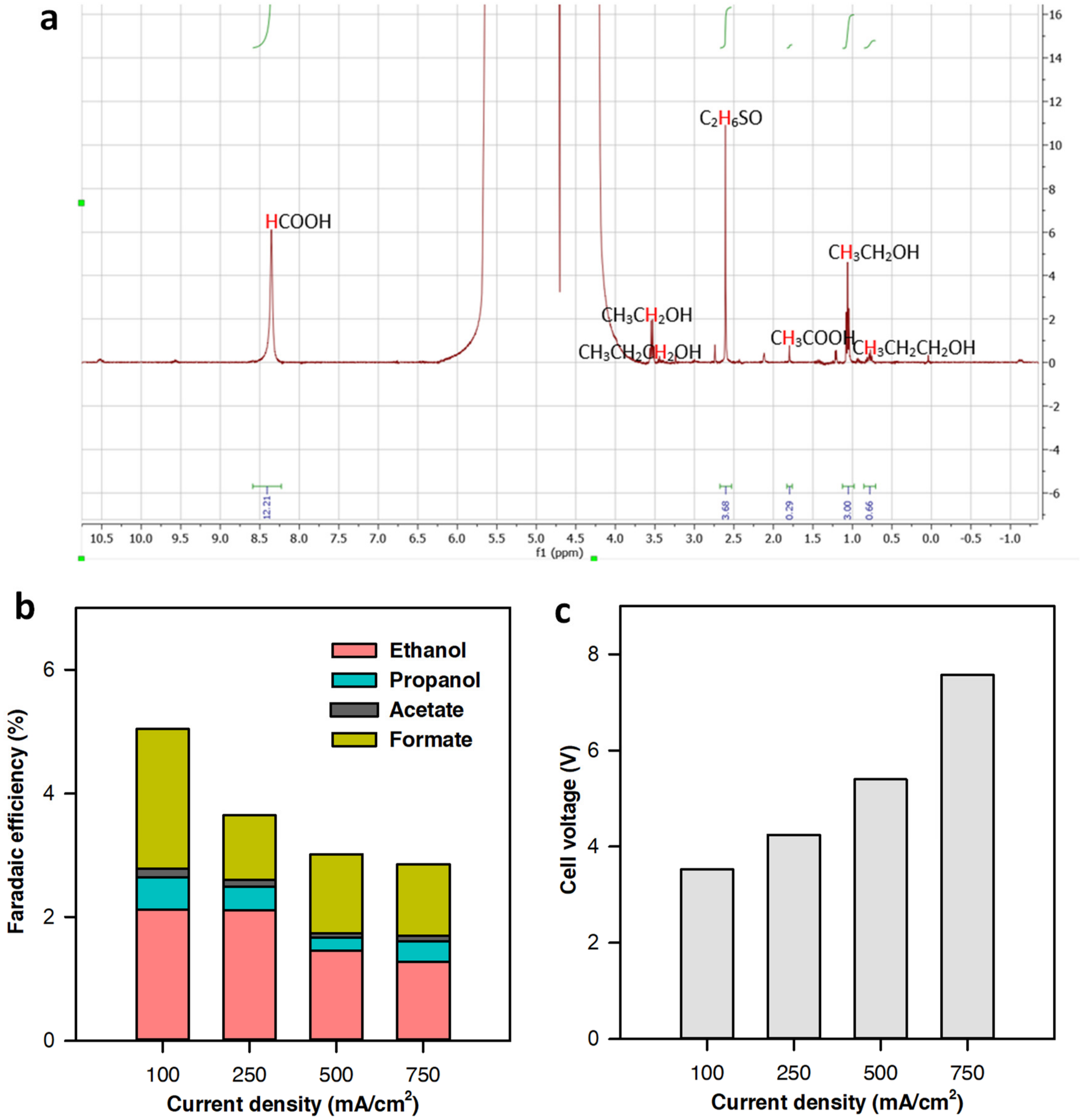

Fig. S6 | Liquid product distribution and cell voltage. A representative ^{1}H-NMR spectrum of the liquid products showing the presence of formate, ethanol, propanol, and acetate (**a**). Liquid product distribution (**b**) and full cell voltage (**c**) at different current densities of Cu mesh operated using alternating negative and positive currents (oxidation current density: 2.5 mA cm^{-2}; oxidation time: 5 s; reduction time: 25 s).

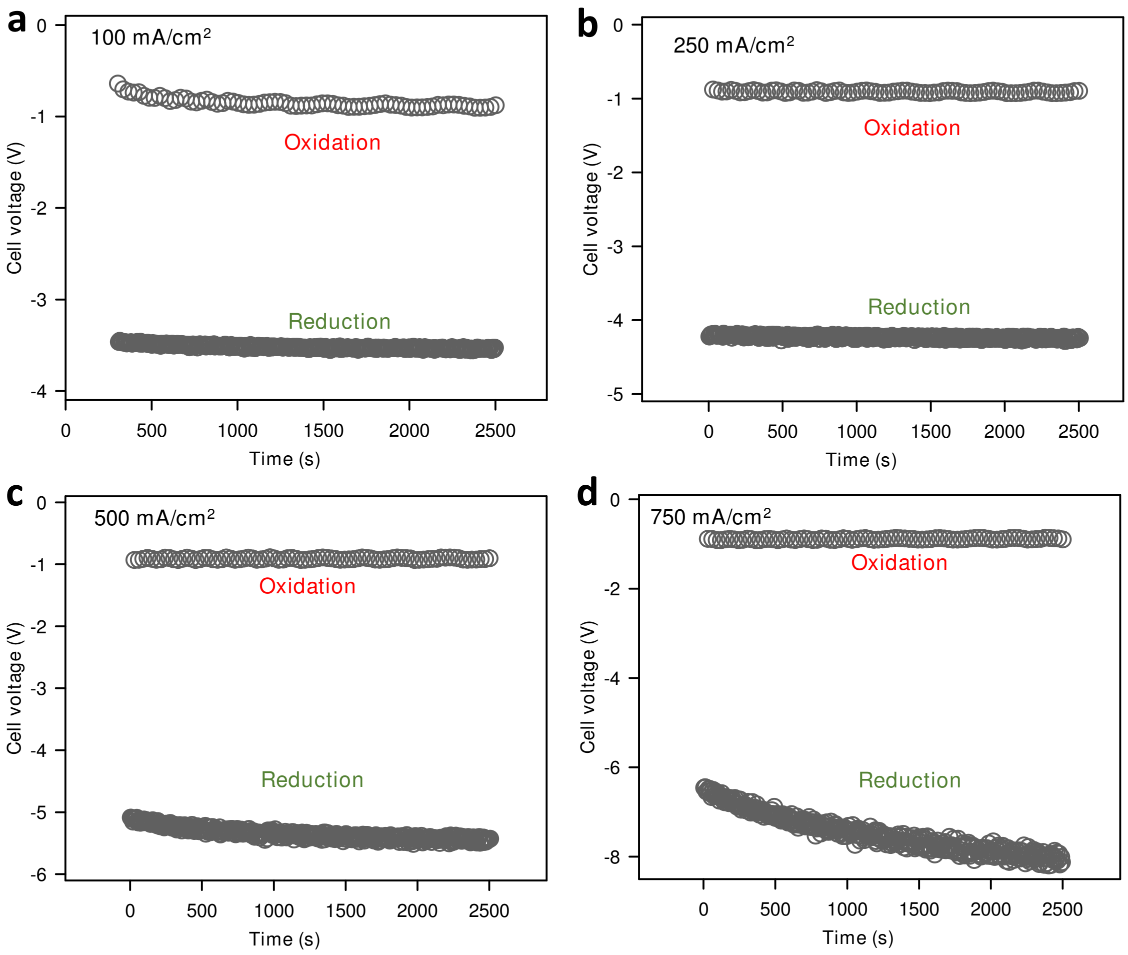

Fig. S7 | Full cell voltage during product screening process at different current densities: 100 mA cm^{-2} (**a**); 250 mA cm^{-2} (**b**); 500 mA cm^{-2} (**c**); and 750 mA cm^{-2} (**d**). The oxidation current density and time were 2.5 mA cm^{-2} and 5 s, respectively. The reduction time was 25 s.

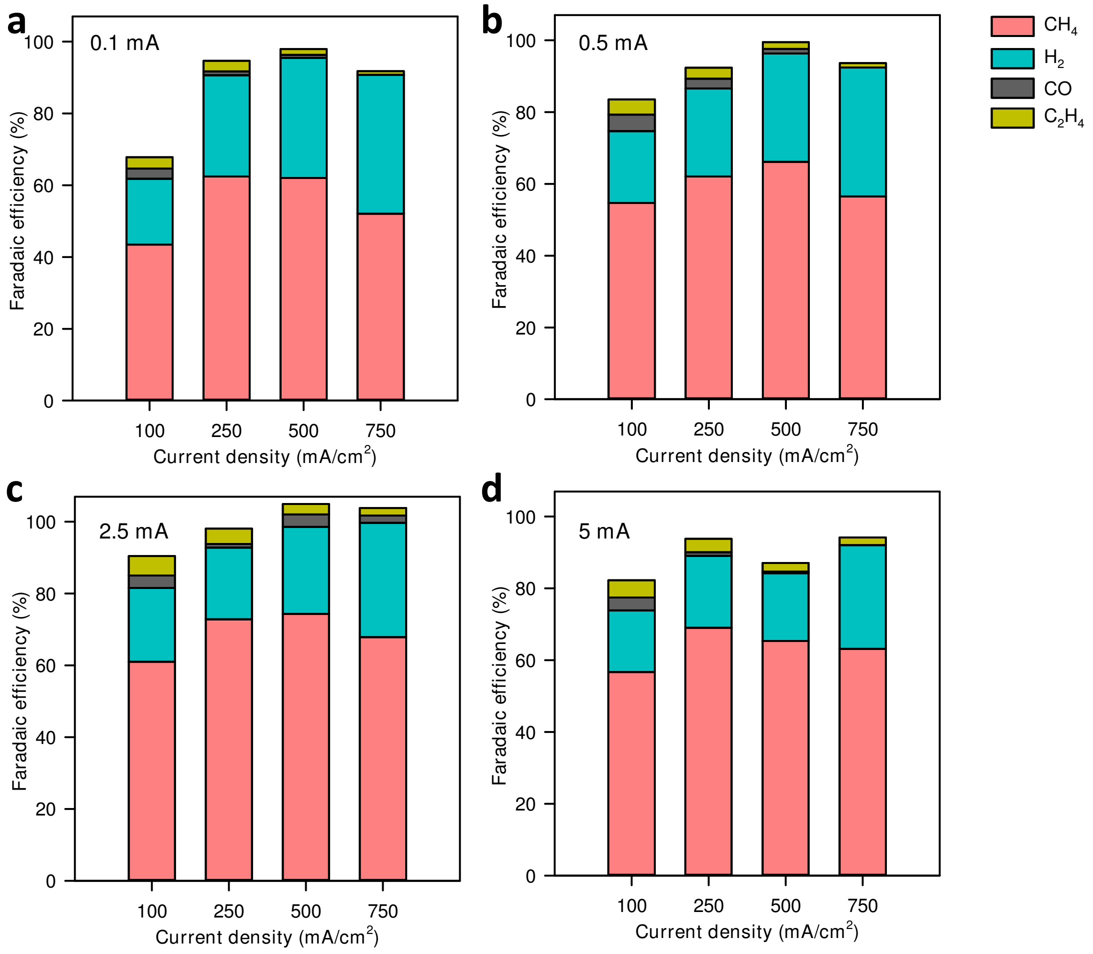

Fig. S8 | Effect of oxidation current. Gas product distribution at different current densities of Cu mesh operated using alternating negative and positive currents. The oxidation current density was 0.1 mA cm^{-2} (**a**); 0.5 mA cm^{-2} (**b**); 2.5 mA cm^{-2} (**c**) and 5 mA cm^{-2} (**d**). The oxidation and reduction times were fixed at 5 s and 25 s, respectively.

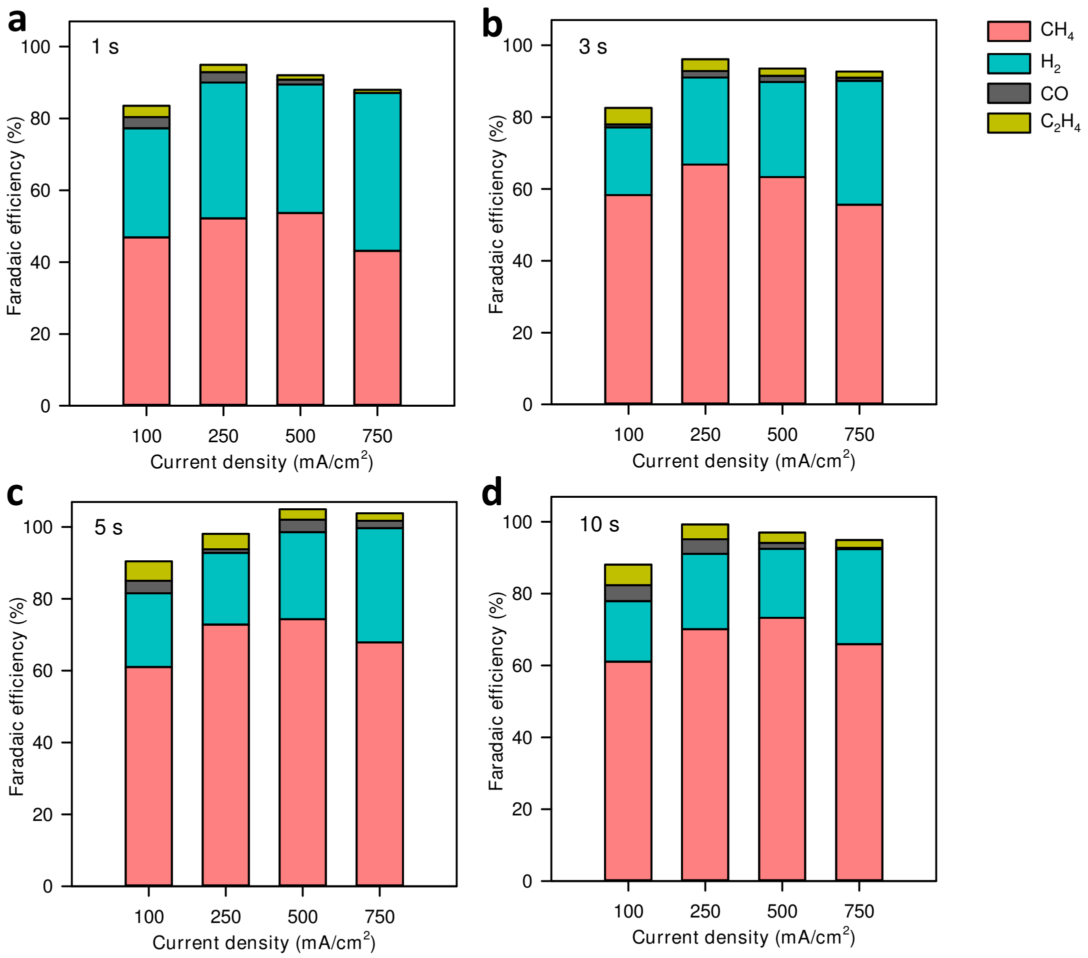

Fig. S9 | Effect of oxidation time. Gas product distribution at different current densities of Cu mesh operated using alternating negative and positive currents. The oxidation times were 1 s (**a**); 3 s (**b**); 5 s (**c**) and 10 s (**d**). The oxidation current density was 2.5 mA cm^{-2} and reduction time was 25 s.

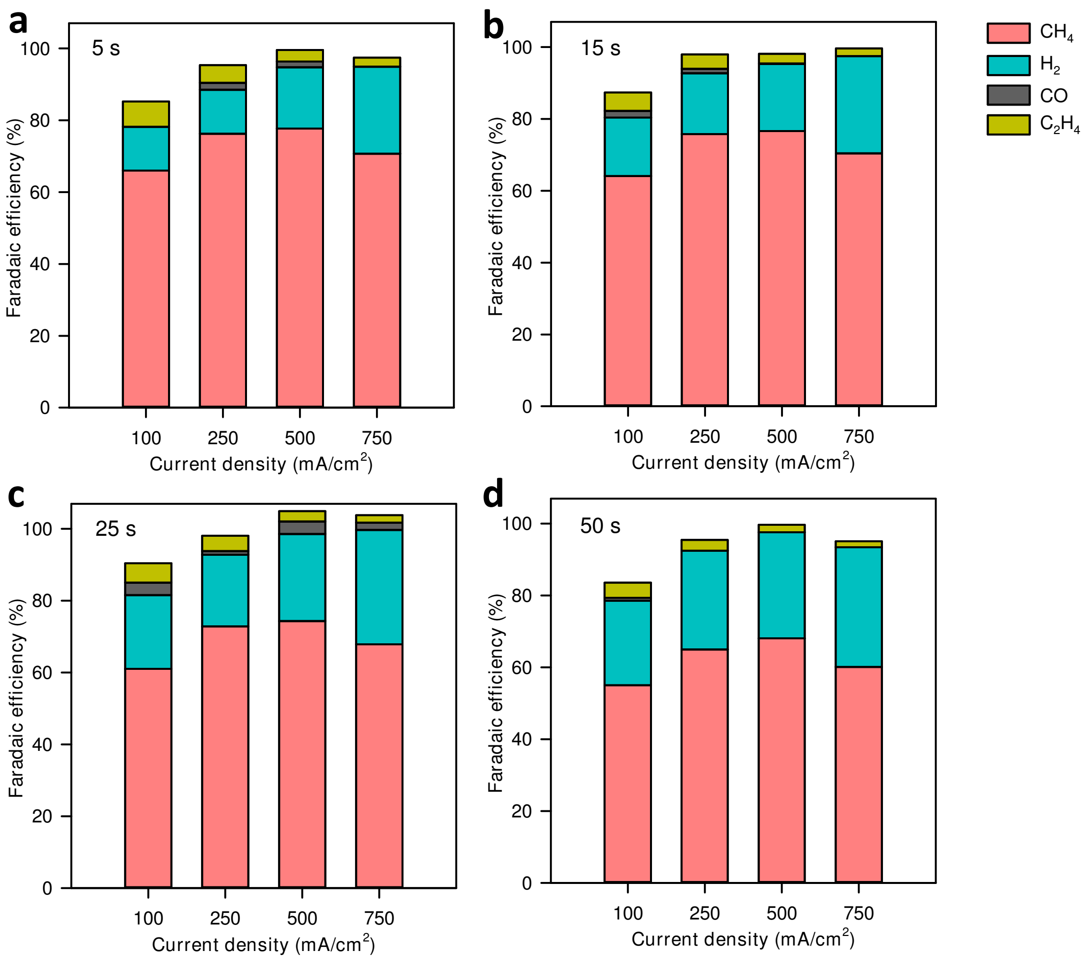

Fig. S10 | Effect of reduction time. Gas product distribution at different current densities of Cu mesh operated using alternating negative and positive currents. The reduction times were 5 s (**a**); 15 s (**b**); 25 s (**c**) and 50 s (**d**). The oxidation current density and time were 2.5 mA cm^{-2} and 5 s, respectively.

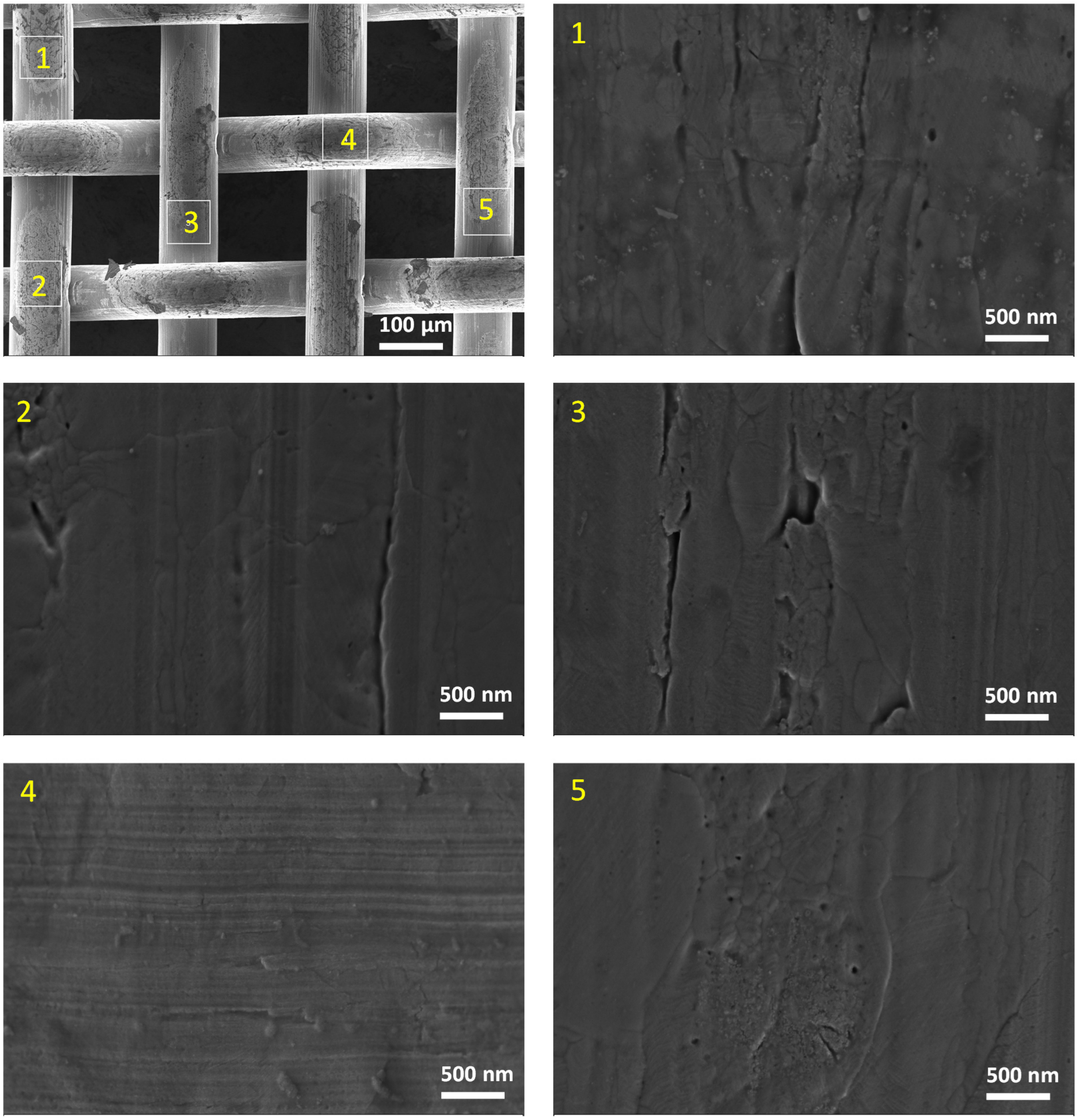

Fig. S11 | SEM images of fresh Cu mesh from multiple locations.

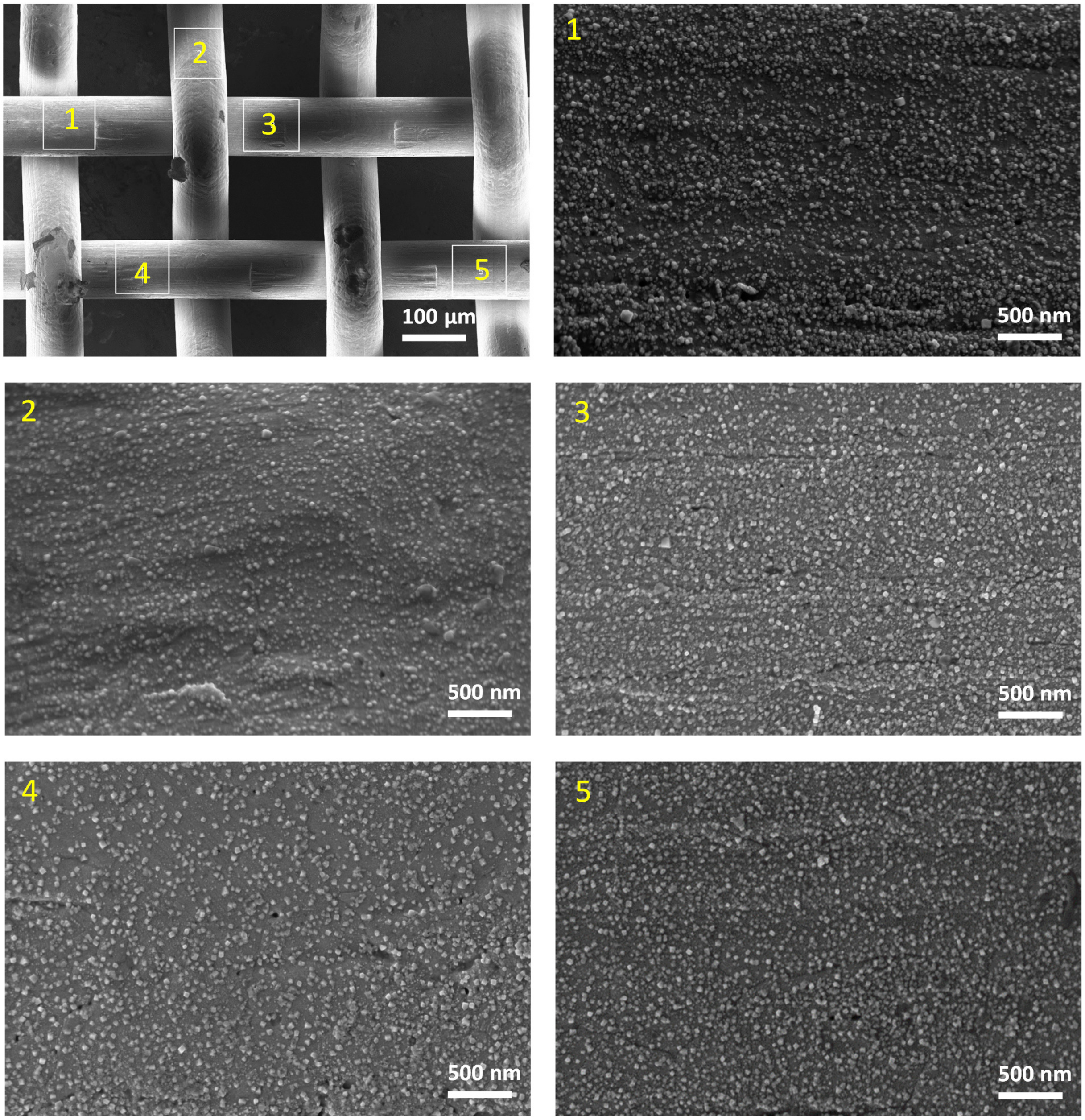

Fig. S12 | SEM images of Cu mesh after constant reduction current operation from multiple locations. The sample for SEM was collected after being tested at 100, 250, 500, and 750 mA cm^{-2} current densities for 40 minutes at each current density (total reaction time of 160 minutes).

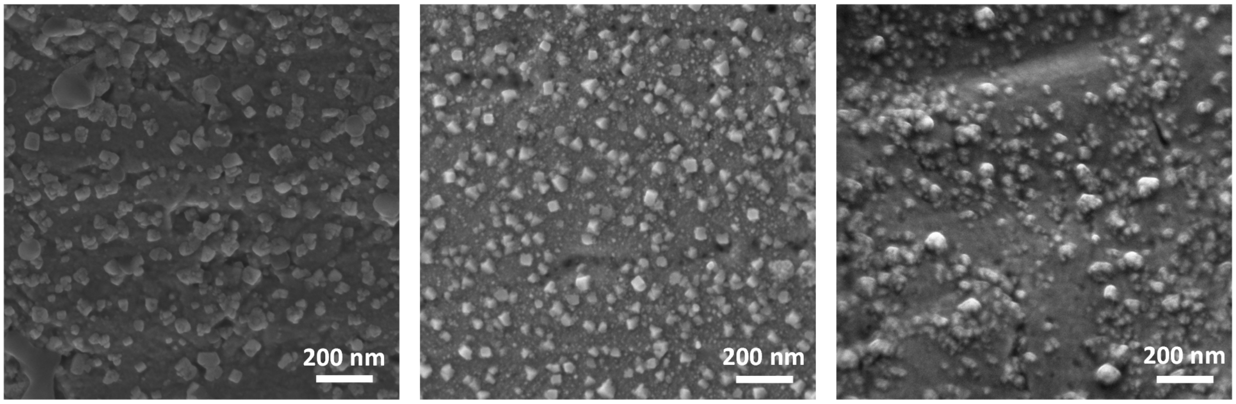

Fig. S13 | SEM images of Cu mesh after constant reduction current operation for three different trials. The sample for SEM was collected after being tested at 100, 250, 500, and 750 mA cm^{-2} current densities for 40 minutes at each current density (total reaction time of 160 minutes).

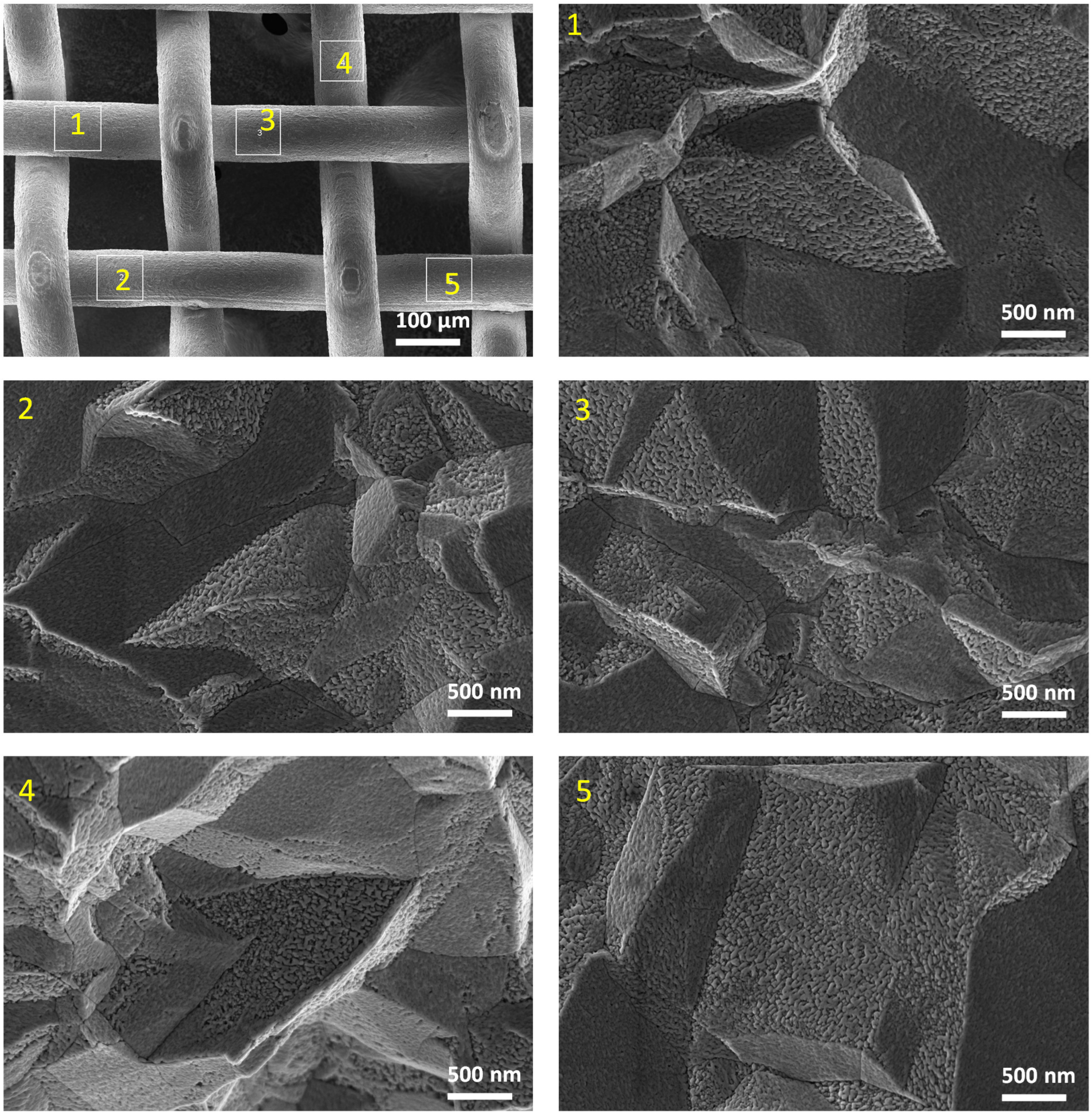

Fig. S14 | SEM images of Cu mesh after alternating current operation from multiple locations. The sample for SEM was collected after being tested at 100, 250, 500, and 750 mA cm^{-2} current densities for 40 minutes at each current density (total reaction time of 160 minutes). The oxidation current density was 2.5 mA cm^{-2}. The oxidation and reduction times were 5 s and 5 s, respectively.

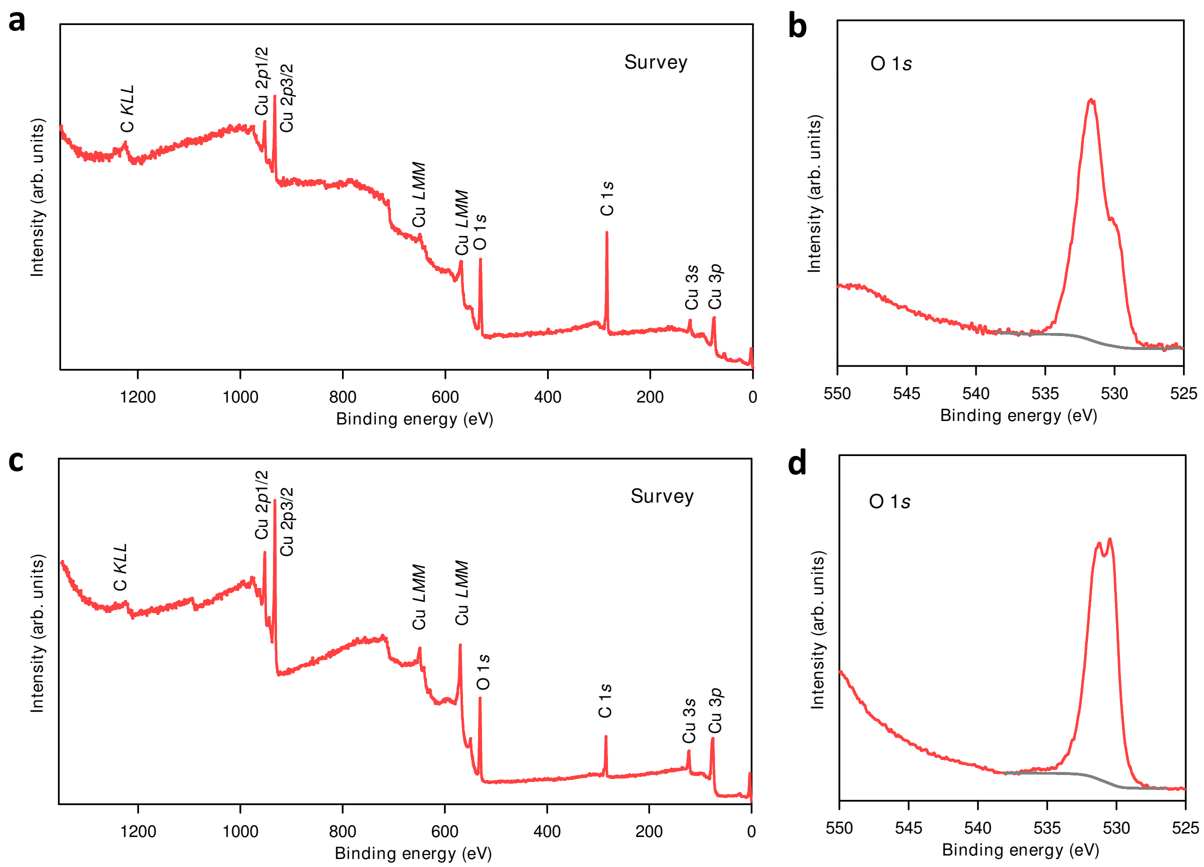

Fig. S15 | XPS spectra of Cu mesh after CO_2 reduction using alternating reduction-oxidation currents (survey (**a**) and O 1*s* (**b**)); and Cu mesh after CO_2 reduction using a fixed current (survey (**c**) and O 1*s* (**d**)). The sample for XPS were collected after being tested at 100, 250, 500, and 750 mA cm^{-2} current densities for 40 minutes at each current density (total reaction time of 160 minutes). For alternating current run, the oxidation current density was 2.5 mA cm^{-2}. The oxidation and reduction times were 5 s and 25 s, respectively. The data show that Cu and O are the two components in the sample after the reaction.

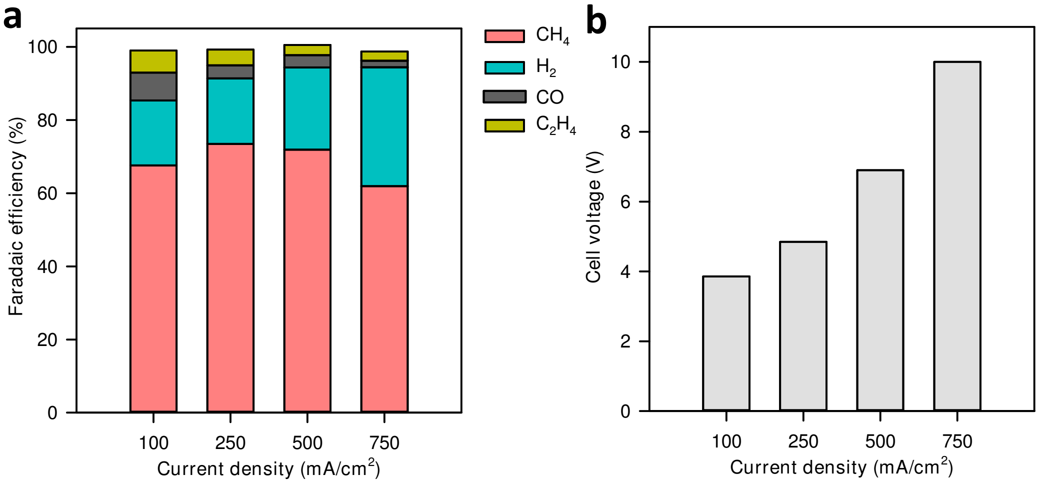

Fig. S16 | Performance with IrO_x/Ti anode. (**a**) CO_2RR product distribution as a function of current density using substitute anode IrO_x/Ti material. (**b**) Cell system voltage as a function of current density. To confirm that the improved selectivity for CH_4 was not due to impurities originating from the use of Ni mesh, we carried out additional experiments where we substituted the Ni mesh with an IrO_x/Ti anode[1, 2]. We observed no significant difference from the use of an alternative anode material in our study. However, we do observe increased cell voltage. (Oxidation current density of 2.5 mA $cm^{-2}$and oxidation time of 5 s; reduction time of 25 s were used for the CO_2 reduction at current density indicated above)

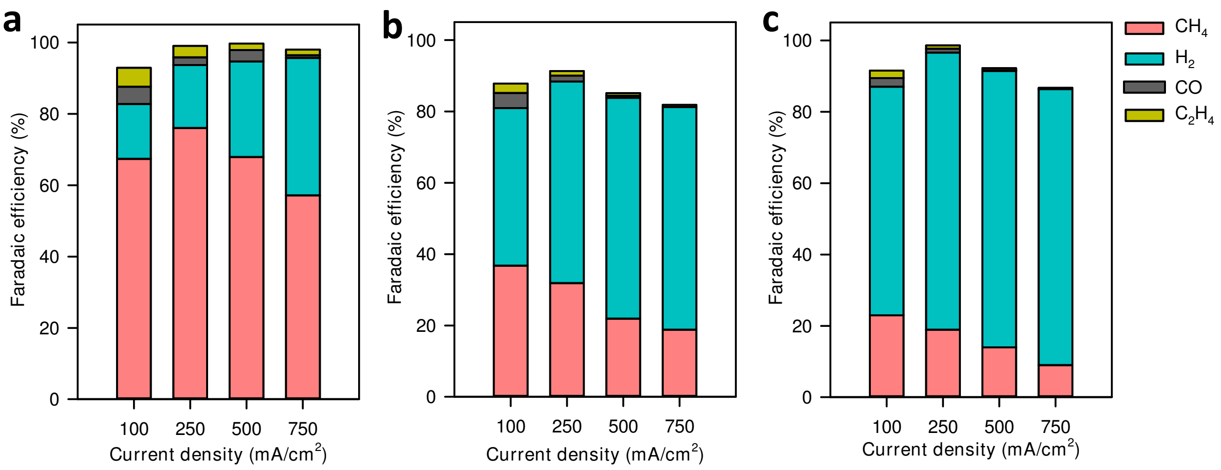

Fig. S17 | Performance after adding Ni impurities. CO_2RR product distribution as a function of current density for (**a**) 0.1 ppm, (**b**) 0.5 ppm, (**c**) 1 ppm concentrations. To exclude the potential effects of Ni on the catalyst performance due to cation migration from the anode side during our alternating current operation conditions we intentionally added Ni cations with different ppm concentrations to the catholyte. With the Ni cations, we observed detrimental effects on CH_4 selectivity at 1 ppm and 0.5 ppm concentrations, while the effect was less significant at 0.1 ppm. (Oxidation current density of 2.5 mA cm^{-2} and oxidation time of 5 s; reduction time of 25 s were used for all conditions)

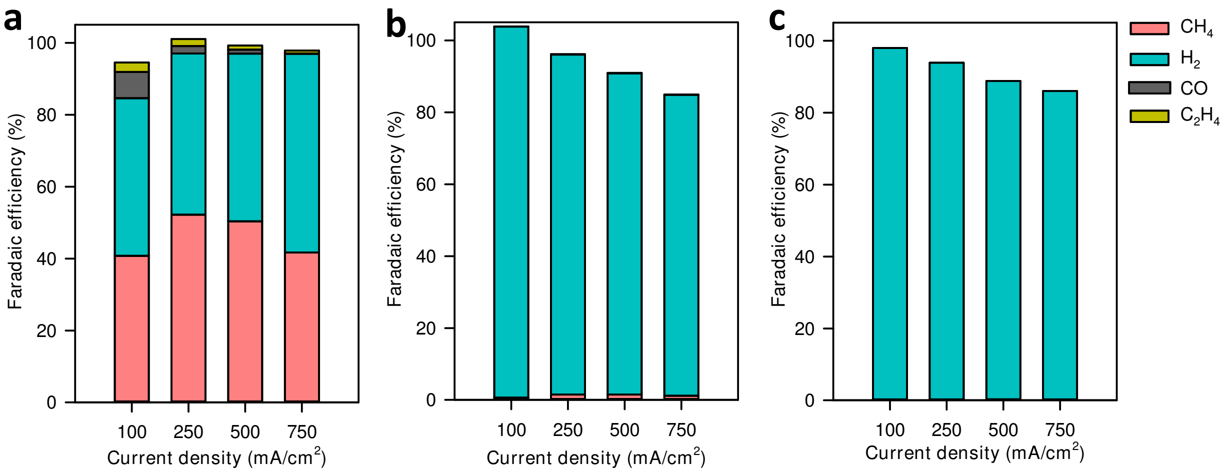

Fig. S18 | Performance after adding Fe impurities. CO_2RR product distribution as a function of current density for (**a**) 0.1 ppm, (**b**) 0.5 ppm, (**c**) 1 ppm concentrations of Fe impurities. To exclude the potential effects of Fe on the catalyst performance due to cation migration from the anode side during our alternating current operation conditions we intentionally added Fe cations with different ppm concentrations to the catholyte. With the Fe cations, we observed severe detrimental effects on CH_4 selectivity at 1 and 0.5 ppm concentrations, and modest detrimental effects at 0.1 ppm. (Oxidation current density of 2.5 mA cm^{-2} and oxidation time of 5 s; reduction time of 25 s were used for all conditions).

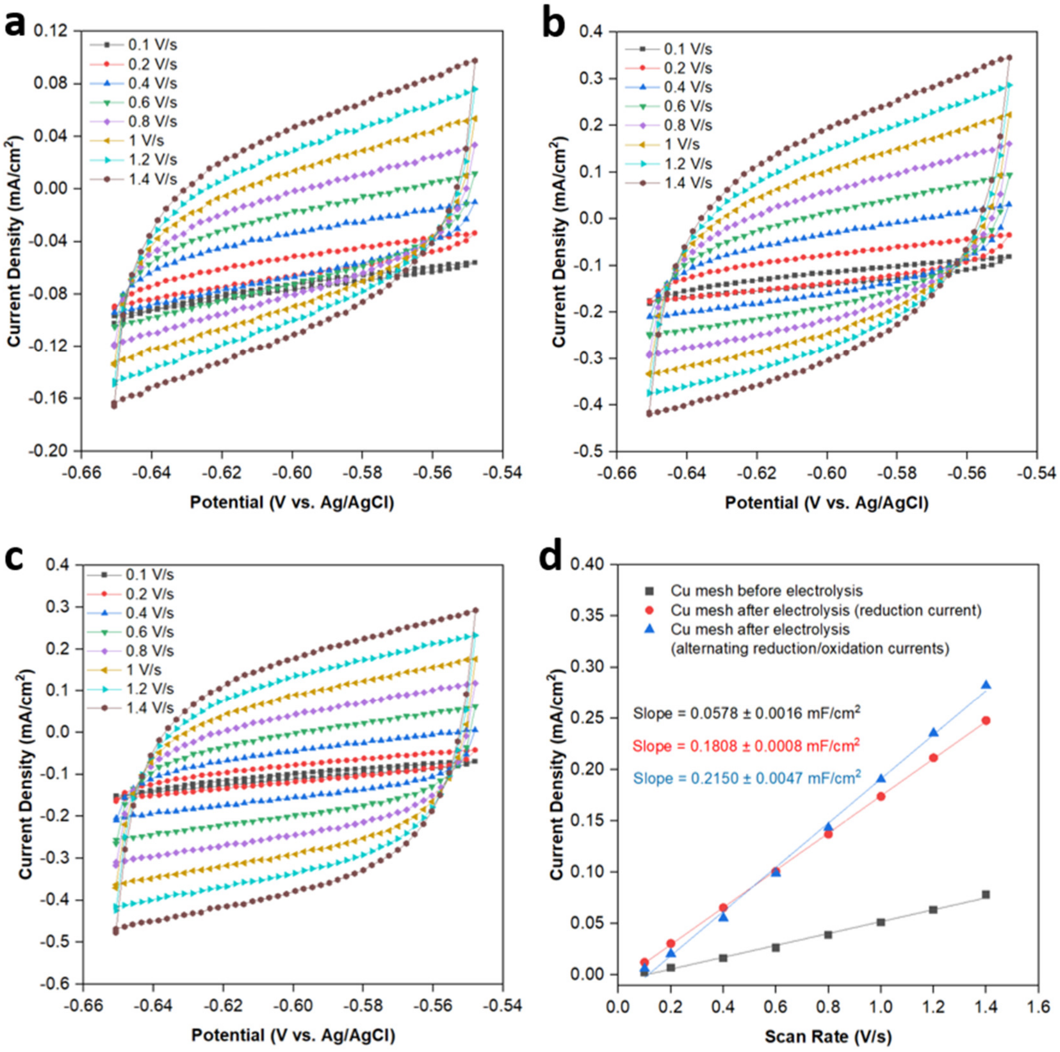

Fig. S19 | Electrochemical double-layer capacitance measurements. Cyclic voltammograms in the non-faradaic region for (**a**) Cu mesh before electrolysis, (**b**) Cu mesh after electrolysis under constant reduction current, and (**c**) Cu mesh after electrolysis under alternating 25 s reduction - 5 s oxidation currents. (**d**) Charging current density plotted as a function of scan rate. The samples (**b** and **c**) were collected after being tested at 100, 250, 500, and 750 mA cm^{-2} current densities for 40 minutes at each current density (total reaction time of 160 minutes). For sample (**c**), the oxidation current density was 2.5 mA cm^{-2}. The oxidation and reduction times were 5 s and 25 s, respectively.

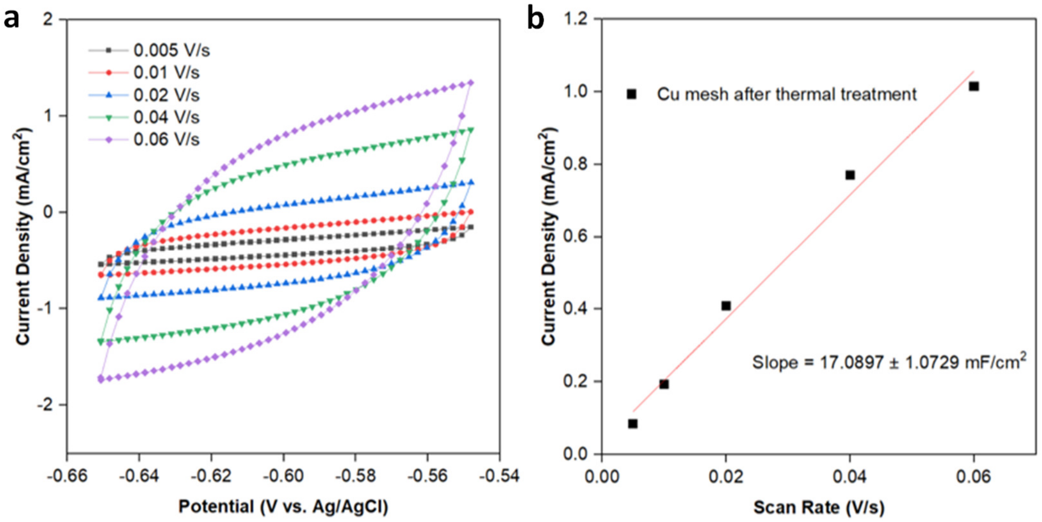

Fig. S20 | Electrochemical double-layer capacitance measurements of thermally treated Cu mesh. (**a**) cyclic voltammograms in the non-faradaic region for Cu mesh after thermal treatment and electrolysis under alternating 25 s reduction – 5 s oxidation currents, and (**b**) the charging current density plotted as a function of scan rate. The sample was collected after being tested at 100, 250, 500, and 750 mA cm^{-2} current densities for 40 minutes at each current density (total reaction time of 160 minutes). The oxidation current density was 2.5 mA cm^{-2}. The oxidation and reduction times were 5 s and 25 s, respectively.

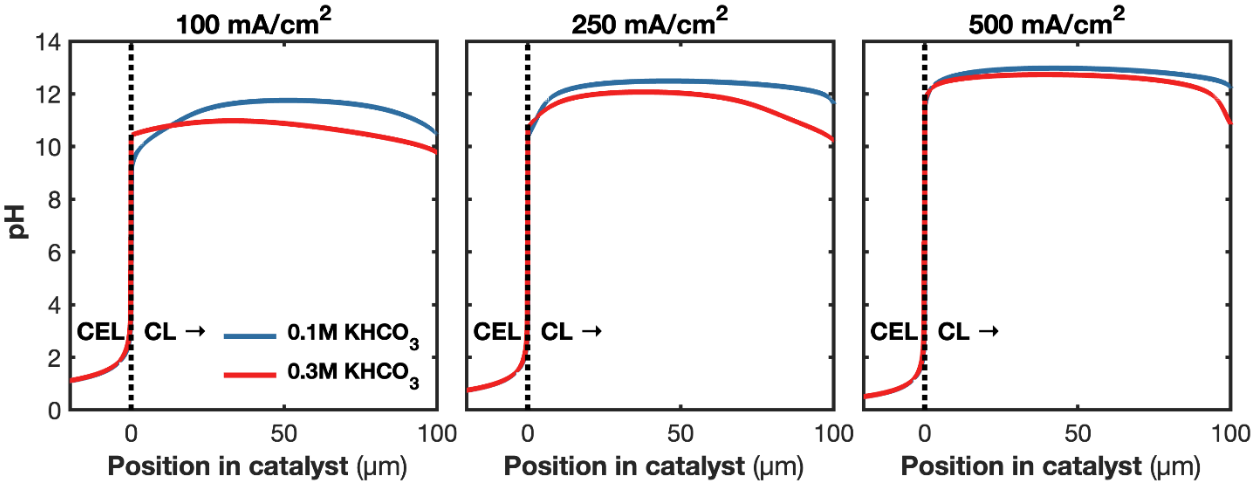

Fig. S21 | Local pH within the catalyst layer. Simulated pH at current densities of 100, 250, and 500 mA cm^{-2} for the open matrix catalyst with a BPM using 0.1 M and 0.3 M $KHCO_3$ catholyte with CO_2 sparging.

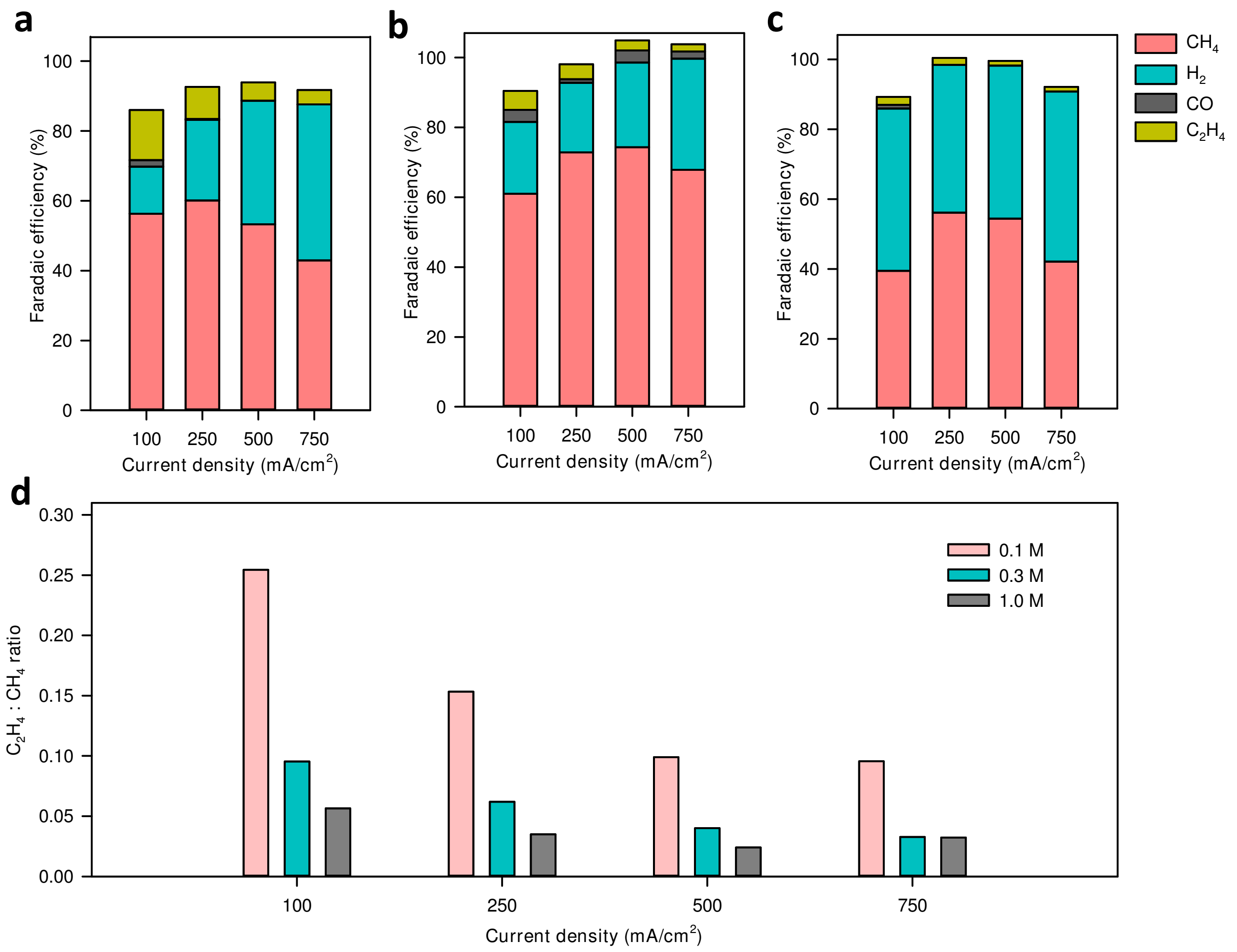

Fig. S22 | Effect of electrolyte concentrations. Gas product distribution at different current densities of Cu mesh operated using alternating negative and positive currents and 0.1 M $KHCO_3$ (**a**); 0.3 M $KHCO_3$ (**b**); and 1 M $KHCO_3$ (**c**). Effect of electrolyte concentration on C_2H_4: CH_4 ratio (**d**). The oxidation current density was 2.5 mA cm^{-2}. The oxidation and reduction times were 5 s and 25 s, respectively.

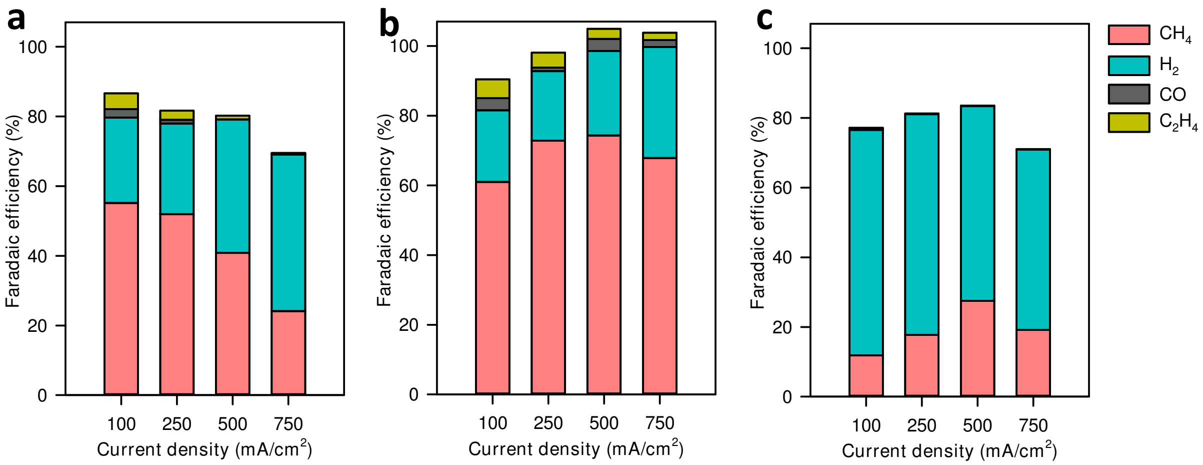

Fig. S23 | Effect of CO_2 sources. Gas product distribution at different current densities of Cu mesh operated using an anion exchange membrane and CO_2 saturated $KHCO_3$ electrolyte (**a**); a bipolar exchange membrane and CO_2 saturated $KHCO_3$ electrolyte (**b**); and a bipolar exchange membrane and N_2 saturated $KHCO_3$ electrolyte (**c**). The oxidation current density was 2.5 mA cm^{-2}. The oxidation and reduction times were 5 s and 25 s, respectively.

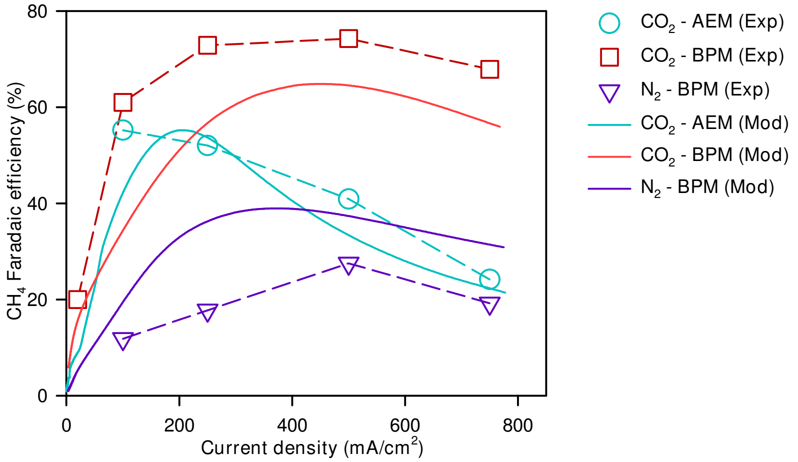

Fig. S24 | Comparison of experimental and modelled ECR performance for various configurations. Experimental CH_4 FE compared with predicted FE from multiphysics modelling for CO_2 and N_2 sparging with a BPM and with an AEM with CO_2 sparging. For the experiments, the oxidation current density was 2.5 mA cm^{-2} and the oxidation and reduction times were 5 s and 25 s, respectively. Catholyte is 0.3 M $KHCO_3$ in experiments and modelling.

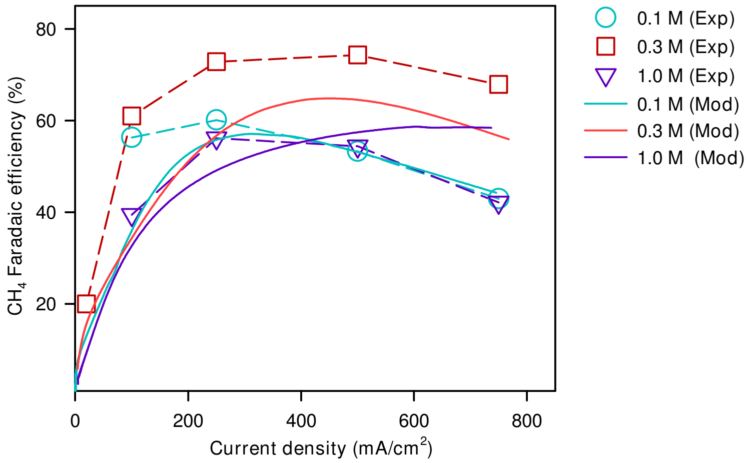

Fig. S25 | Comparison of experimental and modelled ECR performance for various catholyte concentrations. Experimental CH_4 FE compared with predicted FE from multiphysics modelling for various catholyte ($KHCO_3$) concentrations. For the experiments, the oxidation current density was 2.5 mA cm^{-2} and the oxidation and reduction times were 5 s and 25 s, respectively. In all cases a BPM with CO_2 sparging is used.

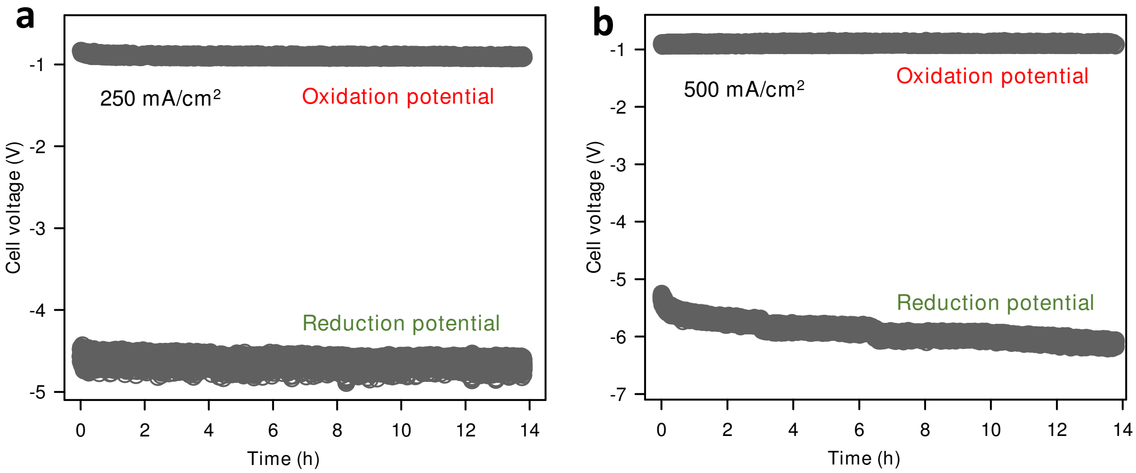

Fig. S26 | Full-cell voltage during stability tests at different current densities: 250 mA cm^{-2} (**a**); and 500 mA cm^{-2} (**b**). The oxidation current density and time were 2.5 mA cm^{-2} and 5 s, respectively. The reduction time was 25 s.

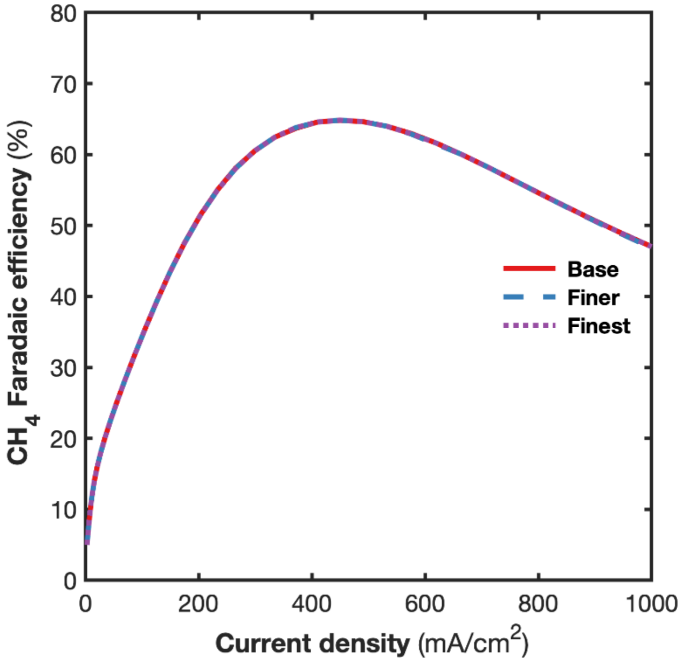

Fig. S27 | Insensitivity of results to numerical resolution. Simulated CH_4 Faradaic efficiency as a function of total current density for 0.3 M $KHCO_3$ with CO_2 sparging at three numerical grid resolutions. 'Finer' is a 2x reduction in numerical grid size compared to the base mesh, and 'finest' is 2x reduction in numerical grid size compared to 'finer.' All lines are coincident.

Table S1. Performance comparison between ECR systems

Catalyst	Cell/Electrolyte	Current (mA/cm^2)	CH_4 FE (%)	J_{CH_4} (mA/cm^2)	Cell Voltage (V)	EE (%)	CH_4 Concentration (%) *	Refs
In-situ activated Cu mesh	Aqueous fed (Neutral)	250	72.9 (72.75)†	182.2	4.25	18.2	16.4	**This work**
In-situ activated Cu mesh	Aqueous fed (Neutral)	500	74.3 (74.22)†	371.6	5.4	14.6	23.4	**This work**
In-situ activated Cu mesh	Aqueous fed (Neutral)	750	67.8 (67.75)†	508.5	7.57	9.5	19.8	**This work**
Cu Foam	Bicarbonate-fed (Neutral)	400	27	120 ± 10	7.2	4.1	8.3**	Ref(1)[3]
Low coordination Cu + CNP additives	MEA	220	62	136	4.0	16.4	1.6	Ref(2)[4]
poly-Cu GDE + 13mM EDTMPA	Flow cell (Alkaline)	300	64 ± 2	192 ± 6	-	-	2.6	Ref(3)[5]
Cu-np/NC	Flow cell (Alkaline)	320	73.4	234	-	-	-	Ref(4)[6]
	MEA	230	60	138	4.0	15.9	5.2	
(Ir1)-doped hybrid Cu_3N/Cu_2O	Flow cell (Alkaline)	320	75	240	-	-	2.3	Ref(5)[7]
2Bn-Cu@UiO-67	Flow cell (Alkaline)	420	81	340	-	-	-	Ref(6)[8]
Ag@Cu2O-6.4 NCs	Flow cell (Alkaline)	250	74 ± 2	178 ± 5	-	-	0.7	Ref(7)[9]
SPVP-Cu NPs	Flow cell (Alkaline)	200	70.64	141	-	-	1.1	Ref(8)[10]
Cu/La2CuO4 Perovskite catalyst	Flow cell (Alkaline)	205	56.3	117	-	-	1.1	Ref(9)[11]
Cu-TDPP-NS	Flow cell (Neutral)	262	70	183	-		0.9	Ref(10)[12]
7% Au-Cu	Flow cell (Neutral)	200	56 ± 2	112 ± 10	-	-	0.2	Ref(11)[13]
Sputtered Cu	Flow cell (Neutral)	225	48 ± 2	108 ± 5	-	-	-	Ref(12)[14]
Sputtered Cu	Flow cell (Neutral)	250	48 ± 4	120 ± 10	-	-	-	Ref(13)[15]
CoO-2.5 nm/Cu/PTFE	Flow cell (Neutral)	225	60 ± 2.2	135	-	-	0.5	Ref(14)[16]

Additional notes:

Table S1: Abbreviations and meanings

EDTMPA – ethylenediamine tetramethylenephosphonic acid

Cu-TDPP-NS - 5, 10, 15, 20-tetrakis(4(2,4-diaminotriazinyl)phenyl)porphyrin Copper nanosheets

SPVP-Cu NPs – Sufficient Polyvinyl Pyrrolidone Copper Nanoparticles

CNP additives – Carbon Nanoparticle additives

Cu-np/NC - Cu nanoparticles supported on N-doped carbon

(*): CH_4 concentration, in the outlet was estimated based on CH_4 FE, total current, electrode area, and CO_2 input flow rate.

CH_4 concentration = $V(CH_4)/V(CO_2) * 100\%$

$V(CO_2)$ = CO_2 flow rate at the outlet of the reactor (mL/min)

$V(CH_4)$ = production rate of CH_4 (mL/min)

$V(CH_4)$ (mL/min) = $FE(CH_4)$ * J (mA/cm^2) * A (cm^2) * 60 (s/min) * 24.47 (mL/mmol) / (8 * 96485 (s* mA/mmol))

In which:

- $FE(CH_4)$ is CH_4 Faradaic efficiency
- J is total current density (mA/cm^2)
- A is total electrode area (cm^2)
- 60 is conversion constant (s/min)
- 24.47 is volume of 1 mmol gas at 25°C
- 8 is number electron transferred in CO_2 reduction to CH_4
- 96485 is Faradaic constant (s * mA/mmol or s*A/mol)

(**): CH_4 concentration was estimated based on the assumption that N_2 is not presented in the gas products and only CH_4, CO_2, and H_2 are presented. According to the paper, $V(CO_2)/V(CH_4)$ = 100/34 = 2.94; $V(H_2)/V(CH_4)$ = 8*$FE(H_2)$/(2*$FE(CH_4)$) = 8*55/2*27 = 8.14. CH_4 concentration = $V(CH_4)/(V(CH_4) + V(CO_2) + V(H_2))$ = 1/12.08 = 8.3%. (V = volume; FE = Faradaic efficiency).

(†): FE determined by considering both reduction and oxidation current.

Table S2. **Model parameters.** Parameters used in the multiphysics model with their corresponding source.

Symbol	Description	Value	Unit	Reference
**Catalyst and membrane layer properties**				
L_{CL}	Catalyst layer width	100	μm	This work
L_{CEL}	Membrane layer width	75	μm	Ref(15)[17]
σ_{CL}	Catalyst layer conductivity	100	S/cm	Ref(16)[17]
D_{fp}	Flow plate channel diameter	0.4	mm	This work
**Transport properties**				
D_{CO3--}	CO_3^{2-} diffusion coefficient	0.923×10^{-9}	m^2/s	Ref(16)[18]
D_{HCO3-}	HCO_3^- diffusion coefficient	1.185×10^{-9}	m^2/s	Ref(16)[18]
D_{H+}	H^+ diffusion coefficient	9.311×10^{-9}	m^2/s	Ref(16)[18]
D_{OH-}	OH^- diffusion coefficient	5.293×10^{-9}	m^2/s	Ref(16)[18]
D_{K+}	K^+ diffusion coefficient	1.957×10^{-9}	m^2/s	Ref(16)[18]
D_{CO2}	CO_2 diffusion coefficient	1.91×10^{-9}	m^2/s	Ref(16)[18]
**Electrochemical reaction rates**				
$i_{0,HER}$	Partial exchange current density, HER reaction	2×10^{-6}	mA/cm^2	Fit
$i_{0,CH4}$	Partial exchange current density, methane reaction	0.5×10^{-9}	mA/cm^2	Fit
α_{HER}	Cathodic transfer coefficient, HER reaction	0.45		Fit
α_{CH4}	Cathodic transfer coefficient, methane reaction	0.5		Fit
$\gamma_{CO2,CH4}$	CO_2 concentration reaction order, methane reaction	1.5		Fit
$\gamma_{pH,CH4,SHE}$	pH dependence on SHE scale, methane reaction	0		Fit
U_{CH4}	Equilibrium potential for methane, vs. RHE	0.17	V	Ref(16)[18]
**Carbonate equilibrium parameters**				
K_1	Equilibrium constant, $CO_2+H_2O \leftrightarrow H^+ + HCO_3^-$	$10^{-6.37}$	mol/L	Ref(16)[18]
K_2	Equilibrium constant, $HCO_3^- \leftrightarrow H^+ + CO_3^{2-}$	$10^{-10.32}$	mol/L	Ref(16)[18]
K_3	Equilibrium constant, $CO_2 + OH^- \leftrightarrow HCO_3^-$	K_1/K_w	L/mol	Ref(16)[18]
K_4	Equilibrium constant, $HCO_3^- + OH^- \leftrightarrow H_2O + CO_3^{2-}$	K_2/K_w	L/mol	Ref(16)[18]
K_w	Equilibrium constant, $H_2O \leftrightarrow H^+ + OH^-$	10^{-14}	mol^2/L^2	Ref(16)[18]
k_1	Forward rate constant, reaction 1	3.71×10^{-2}	1/s	Ref(16)[18]
k_2	Forward rate constant, reaction 2	59.44	1/s	Ref(16)[18]
k_3	Forward rate constant, reaction 3	2.23×10^{3}	L/mol/s	Ref(16)[18]
k_4	Forward rate constant, reaction 4	6.0×10^{9}	L/mol/s	Ref(16)[18]
k_w	Forward rate constant, reaction *w*	1.4×10^{-3}	mol/L/s	Ref(16)[18]
**CO_2 phase transfer parameters**				
H_{CO2}	Henry's constant, CO_2	29	atm/M	Ref(17)[19]
P_g	Gas pressure	1	atm	Assumed

y_{CO2}	Gaseous CO_2 mole fraction	0.01		Assumed
k_{MT}	Mass transfer coefficient, CO_2 phase transfer	100	1/s	Ref(18)[20]
**Bulk electrolyte concentration, 0.3M Nominal $KHCO_3$ CO_2 sparging** (determined from equilibrium constants)				
c_{CO3--}	CO_3^{2-} bulk electrolyte concentration	2.42×10^{-4}	M	Calculated
c_{HCO3-}	HCO_3^- bulk electrolyte concentration	0.267	M	Calculated
c_{H+}	H^+ bulk electrolyte concentration	5.28×10^{-8}	M	Calculated
c_{OH-}	OH^- bulk electrolyte concentration	1.89×10^{-7}	M	Calculated
c_{K+}	K^+ bulk electrolyte concentration	0.267	M	Calculated
c_{CO2}	CO_2 bulk electrolyte concentration	0.033	M	Ref(19)[21]
**Bulk electrolyte concentration, 0.3M Nominal $KHCO_3$ N_2 sparging** (determined from equilibrium constants)				
c_{CO3--}	CO_3^{2-} bulk electrolyte concentration	$6,38\times10^{-3}$	M	Calculated
c_{HCO3-}	HCO_3^- bulk electrolyte concentration	0.292	M	Calculated
c_{H+}	H^+ bulk electrolyte concentration	2.12×10^{-9}	M	Calculated
c_{OH-}	OH^- bulk electrolyte concentration	4.57×10^{-6}	M	Calculated
c_{K+}	K^+ bulk electrolyte concentration	0.305	M	Calculated
c_{CO2}	CO_2 bulk electrolyte concentration	0.0015	M	Ref(19)[21]

References

1. Klaus, S., Cai, Y., Louie, M. W., Trotochaud, L. & Bell, A. T. Effects of Fe electrolyte impurities on $Ni(OH)_2$/NiOOH structure and oxygen evolution activity. *J. Phys. Chem. C*, **119**, 7243-7254 (2015).
2. Klaus, S., Louie, M. W., Trotochaud, L. & Bell, A. T. Role of catalyst preparation on the electrocatalytic activity of $Ni_{1-x}Fe_xOOH$ for the oxygen evolution reaction. *J. Phys. Chem. C*, **119**, 18303-18316 (2015).
3. Lees, E. W. *et al.* Electrolytic methane production from reactive carbon solutions. *ACS Energy Lett.*, **7**, 1712-1718 (2022).
4. Xu, Y. *et al.* Low coordination number copper catalysts for electrochemical CO_2 methanation in a membrane electrode assembly. *Nat. Commun.*, **12**, 2932 (2021).
5. Han, Z. *et al.* Steering surface reconstruction of copper with electrolyte additives for CO_2 electroreduction. *Nat. Commun.*, **13**, 3158 (2022).
6. Wu, Y. *et al.* Enhancing CO_2 electroreduction to CH_4 over Cu nanoparticles supported on N-doped carbon. *Chem. Sci.* **13**, 8388-8394 (2022).
7. Chen, S. *et al.* Engineering water molecules activation center on multisite electrocatalysts for enhanced CO_2 methanation. *J. Am. Chem. Soc.*, **144**, 12807-12815 (2022).
8. Chen, S. *et al.* MOF encapsulating N-heterocyclic carbene-ligated copper single-atom site catalyst towards efficient methane electrosynthesis. *Angew. Chem. Int. Ed.*, **61**, e202114450 (2022).
9. Xiong, L. *et al.* Geometric modulation of local CO flux in Ag@Cu_2O nanoreactors for steering the CO_2RR pathway toward high-efficacy methane production. *Adv. Mater.*, **33**, 2101741 (2021).
10. Fan, Q. *et al.* Manipulating Cu nanoparticle surface oxidation states tunes catalytic selectivity toward CH_4 or C_{2+} products in CO_2 electroreduction. *Adv. Energy Mater.*, **11**, 2101424 (2021).

11. Chen, S. *et al.* Highly selective carbon dioxide electroreduction on structure-evolved copper perovskite oxide toward methane production. *ACS Catal.*, **10**, 4640-4646 (2020).
12. Wang, Y.-R. *et al.* Implanting numerous hydrogen-bonding networks in a Cu-porphyrin-based nanosheet to boost ch_4 selectivity in neutral-media CO_2 electroreduction. *Angew. Chem. Int. Ed.*, **60**, 21952-21958 (2021).
13. Wang, X. *et al.* Gold-in-copper at low *CO coverage enables efficient electromethanation of CO_2. *Nat. Commun.*, **12**, 3387 (2021).
14. Wang, X. *et al.* Efficient methane electrosynthesis enabled by tuning local CO_2 availability. *J. Am. Chem. Soc.*, **142**, 3525-3531 (2020).
15. Sedighian Rasouli, A. *et al.* CO_2 electroreduction to methane at production rates exceeding 100 mA/cm^2. *ACS Sustain. Chem. Eng.,* **8**, 14668-14673 (2020).
16. Li, Y. *et al.* Promoting CO_2 methanation via ligand-stabilized metal oxide clusters as hydrogen-donating motifs. *Nat. Commun.*, **11**, 6190 (2020).
17. Lees, E. W., Bui, J. C., Song, D., Weber, A. Z. & Berlinguette, C. P. Continuum model to define the chemistry and mass transfer in a bicarbonate electrolyzer. *ACS Energy Lett.*, **7**, 834-842 (2022).
18. Weng, L.-C., Bell, A. T. & Weber, A. Z. Modeling gas-diffusion electrodes for CO_2 reduction. *Phys. Chem. Chem. Phys.*, **20**, 16973-16984 (2018).
19. Enick, R. M. & Klara, S. M. CO_2 solubility in water and brine under reservoir conditions. *Chem. Eng. Commun.*, **90**, 23-33 (1990).
20. Kas, R. *et al.* Modeling the local environment within porous electrode during electrochemical reduction of bicarbonate. *Ind. Eng. Chem. Res.,* **61**, 10461 - 10473 (2022).
21. Min, X. & Kanan, M. W. Pd-catalyzed electrohydrogenation of carbon dioxide to formate: High mass activity at low overpotential and identification of the deactivation pathway. *J. Am. Chem. Soc.*, **137**, 4701-4708 (2015).
